# Supplementary material for: Carbon nanotubes allow capture of krypton, barium and lead for multichannel biological X-ray fluorescence imaging
Source: Nat Commun. 2016 Oct 26;7:13118. doi: 10.1038/ncomms13118 (PMC5095174; doi:10.1038/ncomms13118)
Supplement: Supplementary Information — Supplementary Figures 1-52, Supplementary Tables 1-5, Supplementary Discussion, Supplementary Methods and Supplementary References. [file ncomms13118-s1.pdf]

Supplementary Figures

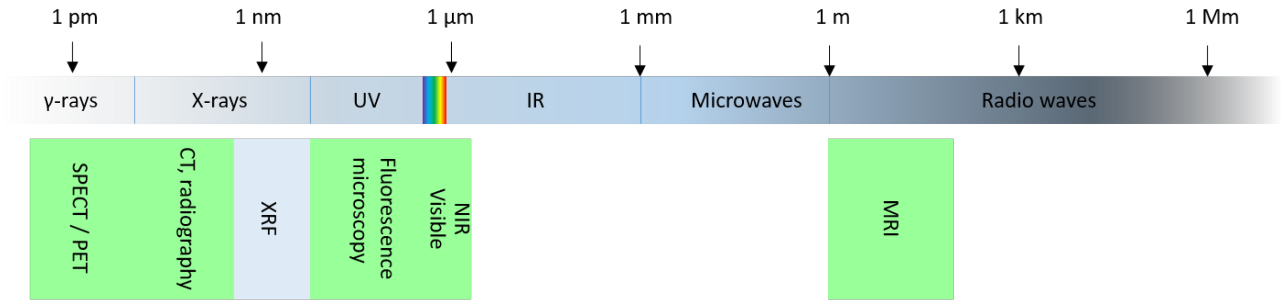

Supplementary Figure 1. Use of the electromagnetic spectrum in medical/biological imaging.

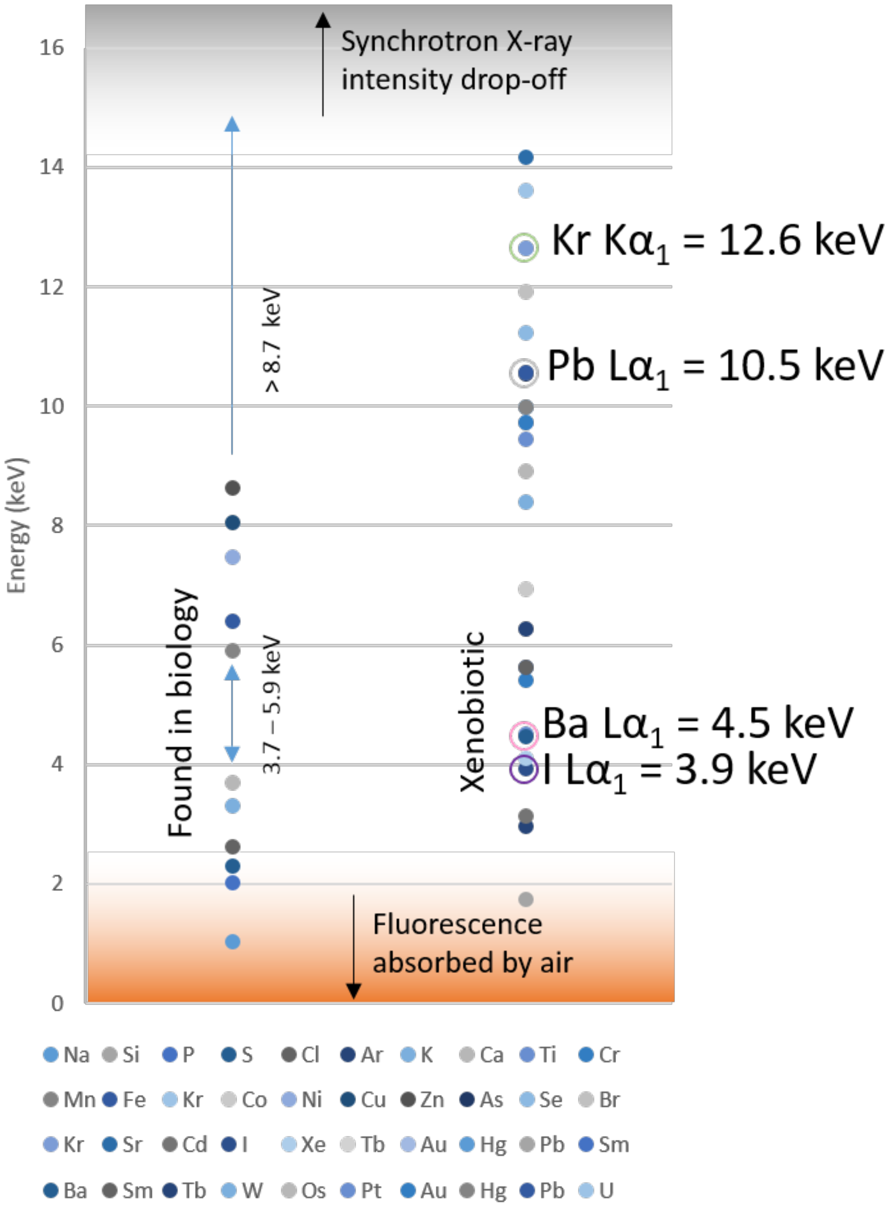

Supplementary Figure 2. Limiting factors for choice of XRF contrast tag elements. Upper energy bound is determined by ability to excite the element using typical synchrotron radiation at XRF beamlines. Lower bound is a result of the absorption of emitted X-rays by air (i.e. nitrogen, oxygen, and carbon). Primary XRF emission lines of biologically prevalent elements are shown on the left, and those of xenobiotic elements on the right. Elements used in this study are highlighted.

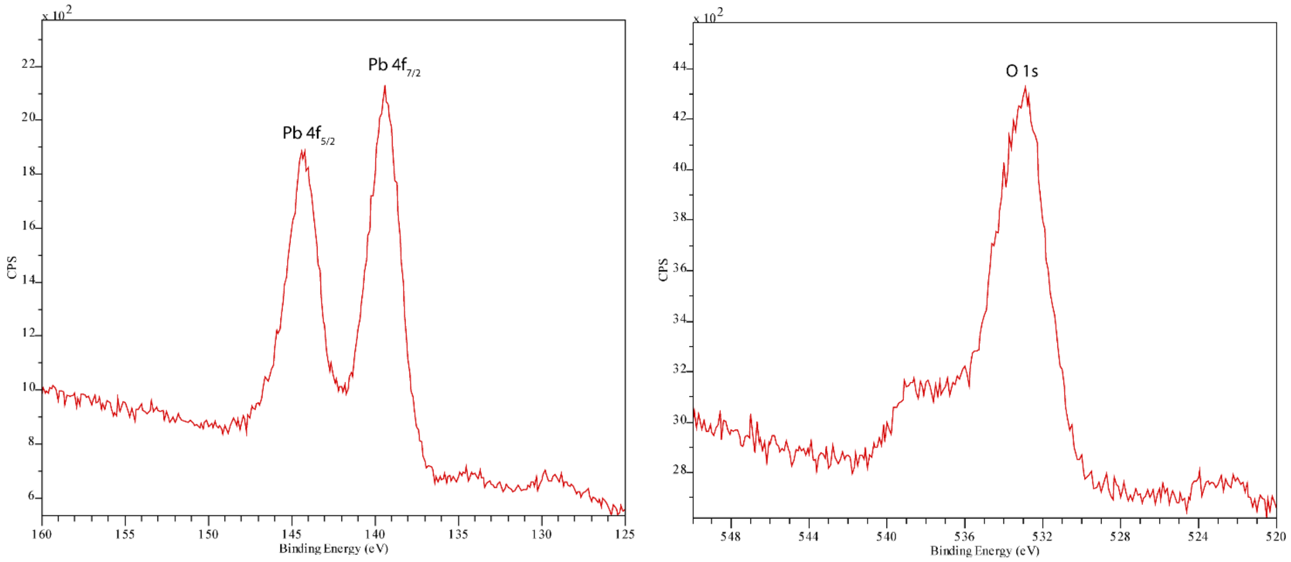

Supplementary Figure 3. XPS spectrum of PbO@SWCNT. Both locations of bands, and the shoulder on the O 1s peak, are consistent with the reported XPS spectrum of α-PbO.[1]

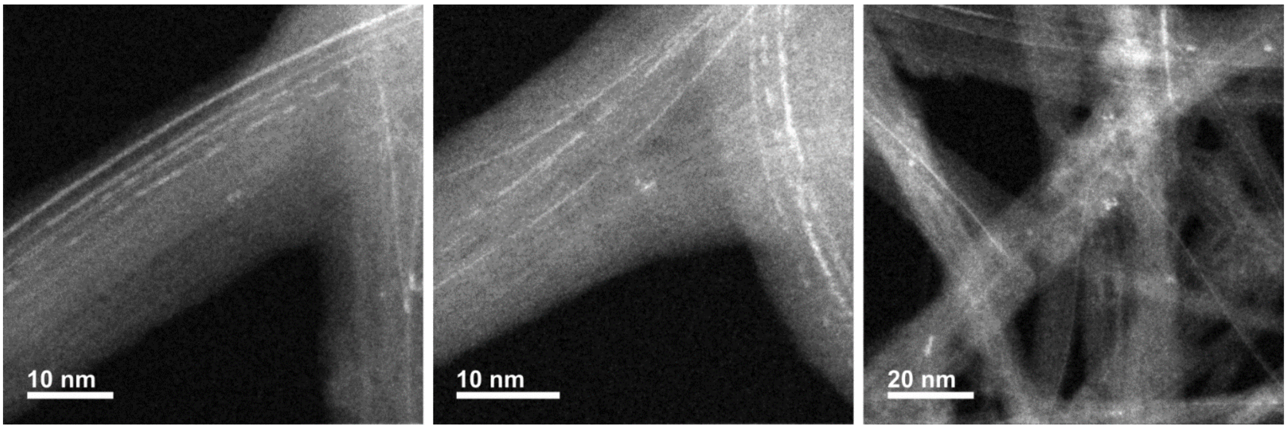

Supplementary Figure 4. Further HAADF STEM images of PbO@SWCNT.

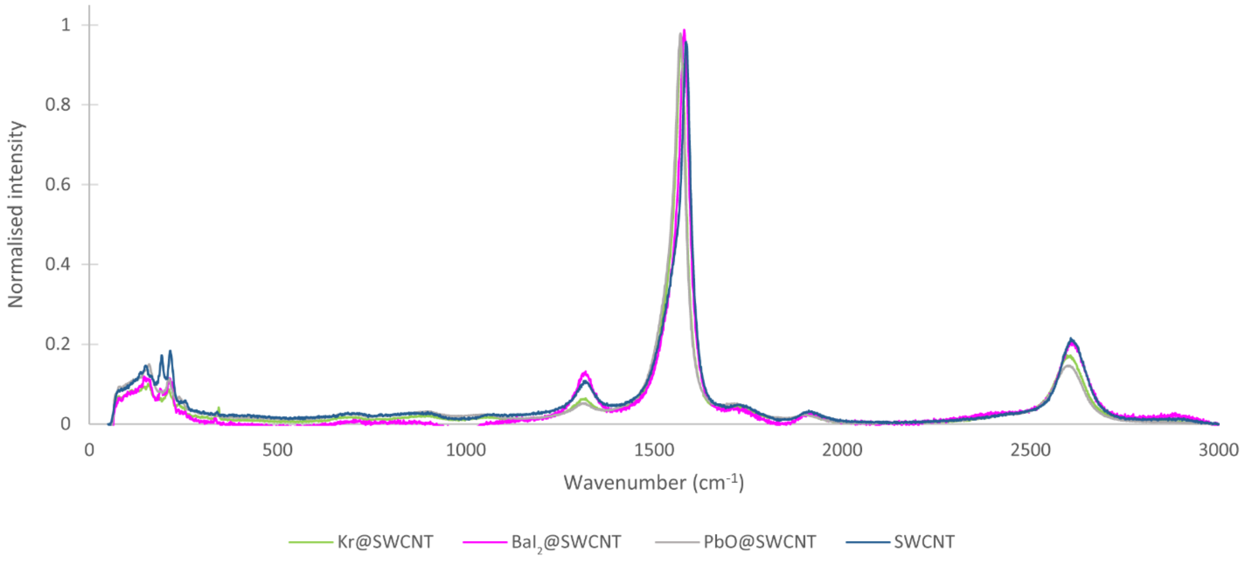

Supplementary Figure 5. Normalised Raman spectra of SWCNT starting material, PbO@SWCNT, BaI_2@SWCNT, and Kr@SWCNT.

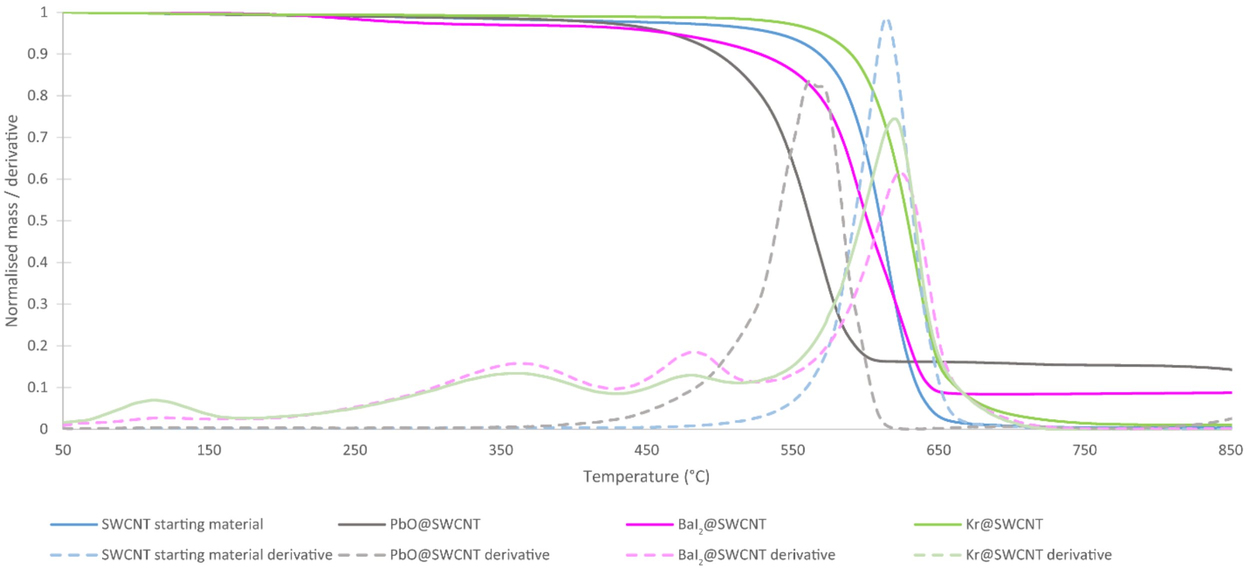

Supplementary Figure 6. TGA curves of SWCNT starting material, PbO@SWCNT, BaI_2@SWCNT, and Kr@SWCNT measured in air.

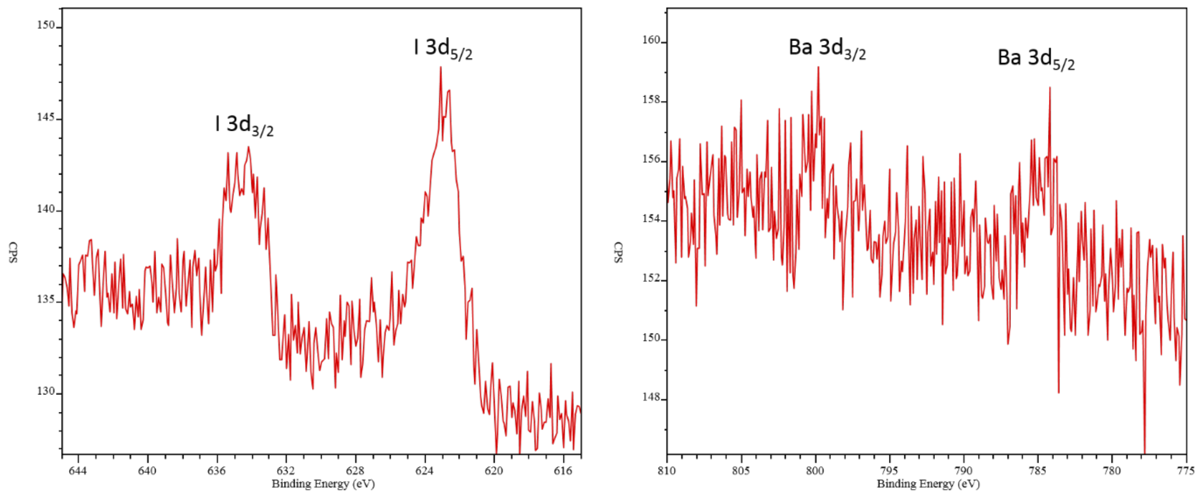

Supplementary Figure 7. XPS spectra of BaI_2@SWCNT.

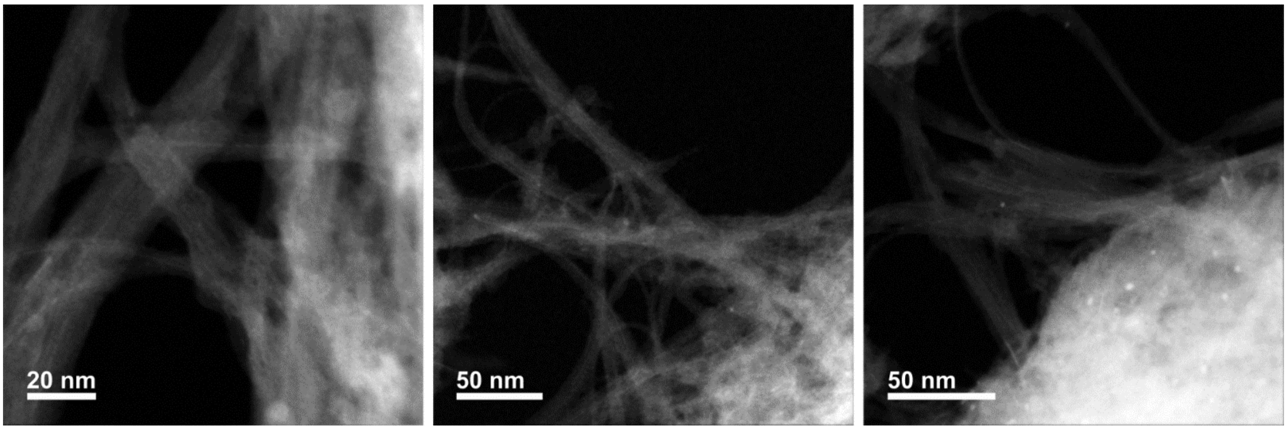

Supplementary Figure 8. Further HAADF STEM images of BaI_2@SWCNT.

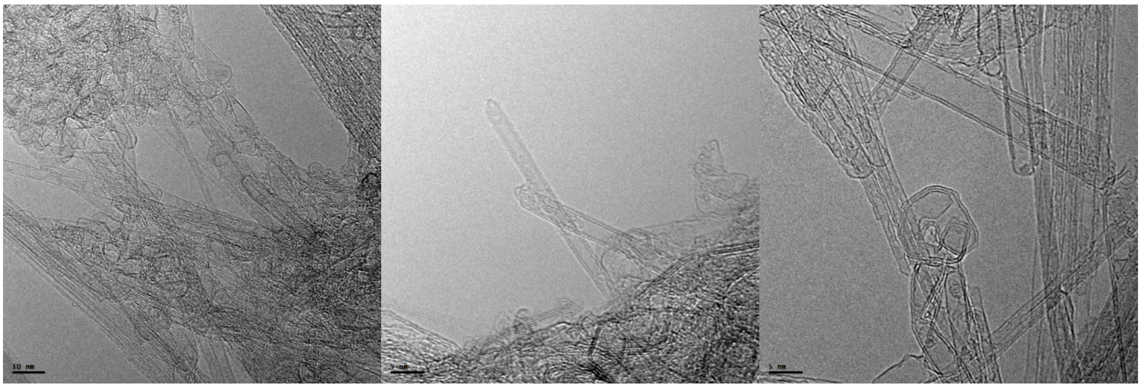

Supplementary Figure 9. Further TEM images of Kr@SWCNT illustrating the closed ends.

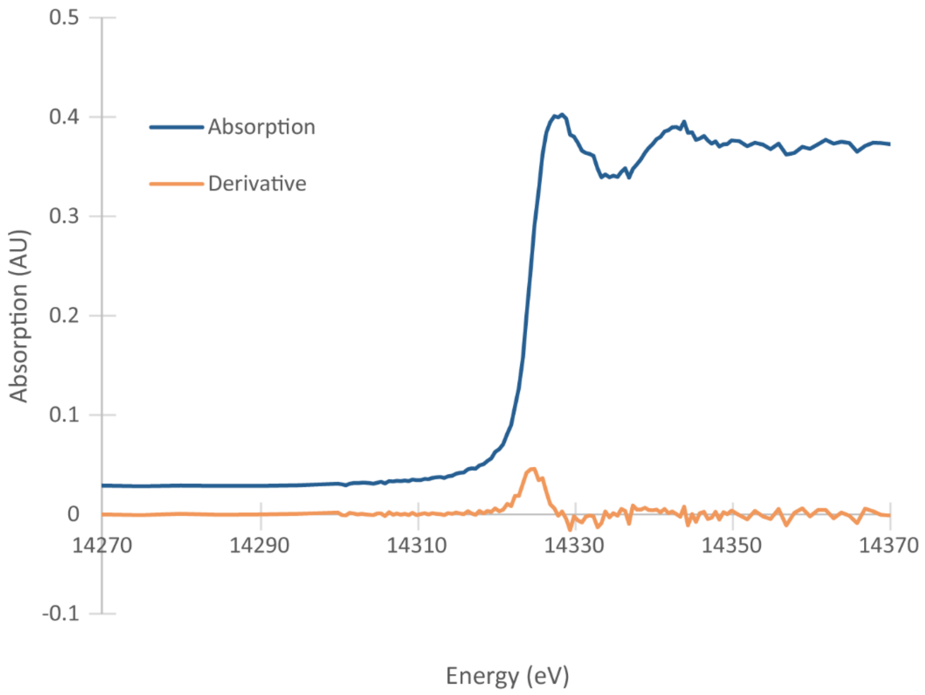

Supplementary Figure 10. X-ray absorption edge of Kr@SWCNT, measured using synchrotron radiation, confirming the elemental assignment. Measured: 14.325 keV, expected: 14.3256 keV.

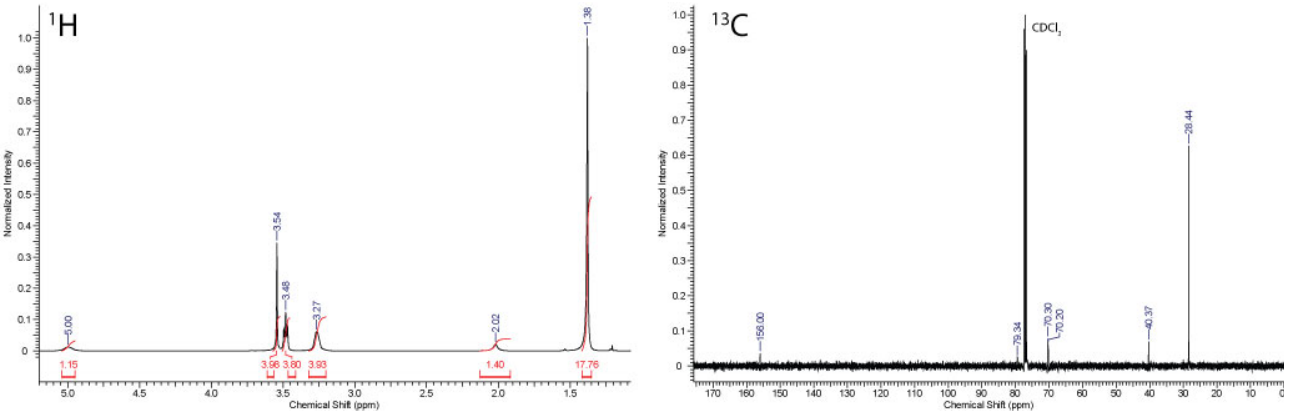

Supplementary Figure 11. ^{1}H and ^{13}C NMR spectra of **1** recorded in $CDCl_3$ (400 MHz).

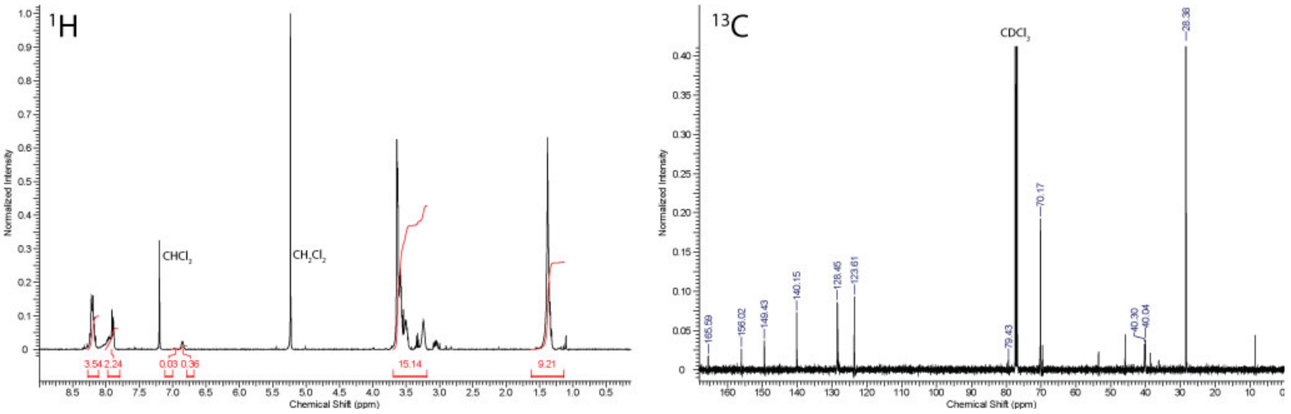

Supplementary Figure 12. ^{1}H and ^{13}C NMR spectra of **2** recorded in $CDCl_3$ (400 MHz).

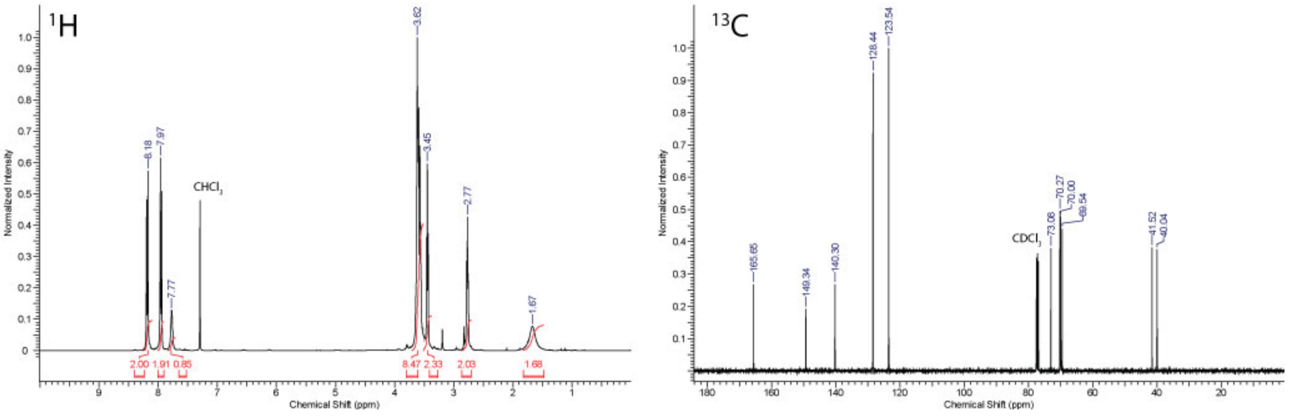

Supplementary Figure 13. ^{1}H and ^{13}C NMR spectra of **3** recorded in $CDCl_3$ (400 MHz).

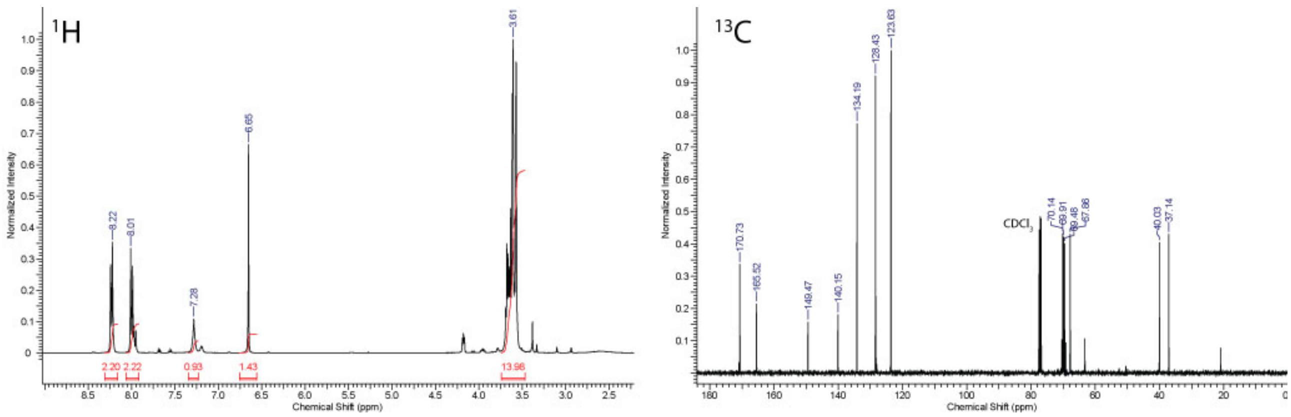

Supplementary Figure 14. ^{1}H and ^{13}C NMR spectra of **4** recorded in $CDCl_3$ (400 MHz).

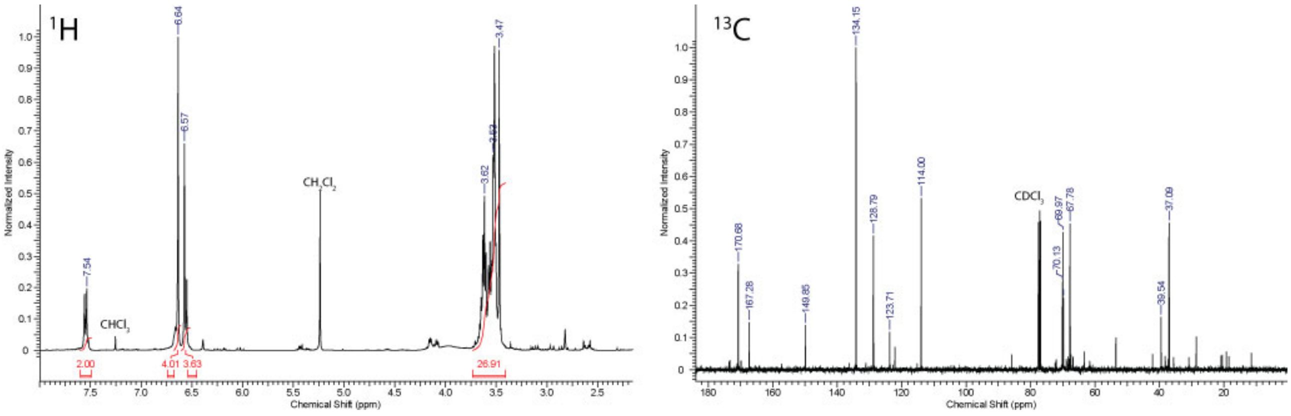

Supplementary Figure 15. ^{1}H and ^{13}C NMR spectra of **5** recorded in $CDCl_3$ (400 MHz).

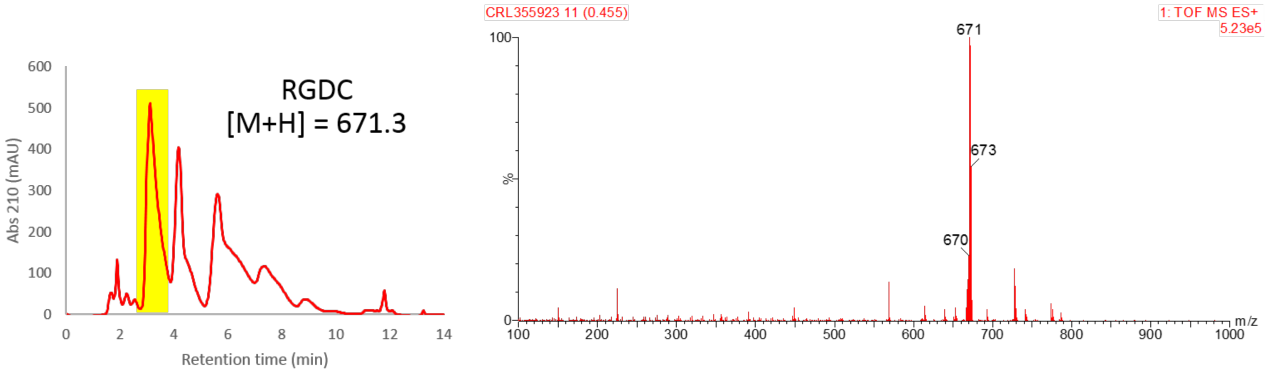

Supplementary Figure 16. HPLC trace and MS spectrum of FmocNH-RGDC-CONH$_2$ peptide for attachment to CNTs. Yellow region in designates portion of the sample collected.

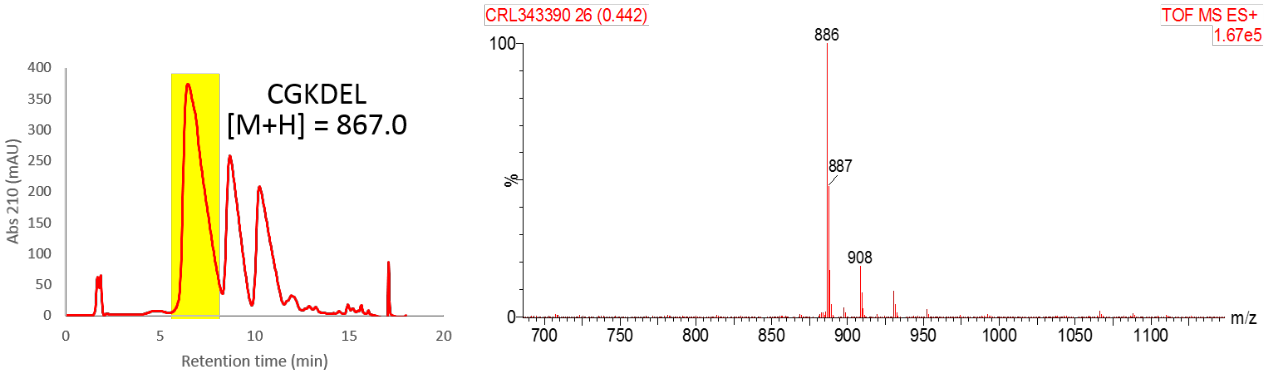

Supplementary Figure 17. HPLC trace and MS spectrum of FmocNH-CGKDEL-CO$_2$H peptide for attachment to CNTs. Yellow region in designates portion of the sample collected.

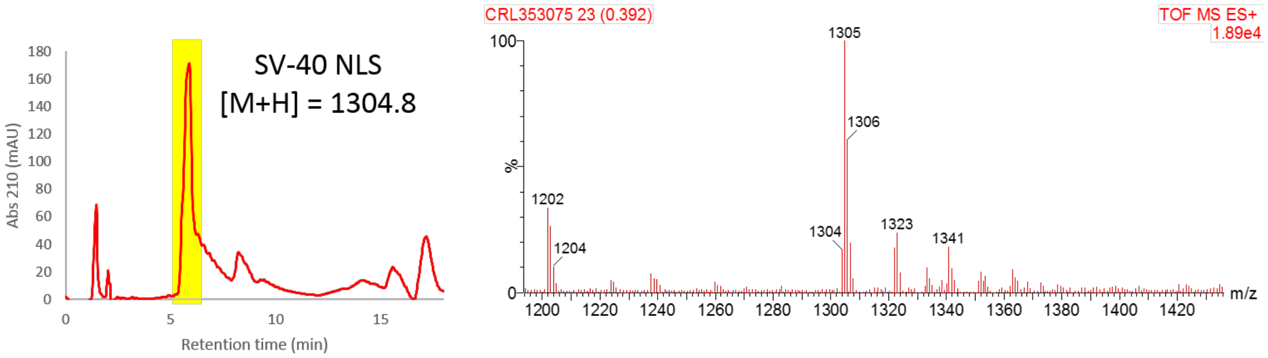

Supplementary Figure 18. HPLC trace and MS spectrum of FmocNH-PPKKKRKVC-CONH$_2$ (SV40 NLS) peptide for attachment to CNTs. Yellow region in designates portion of the sample collected.

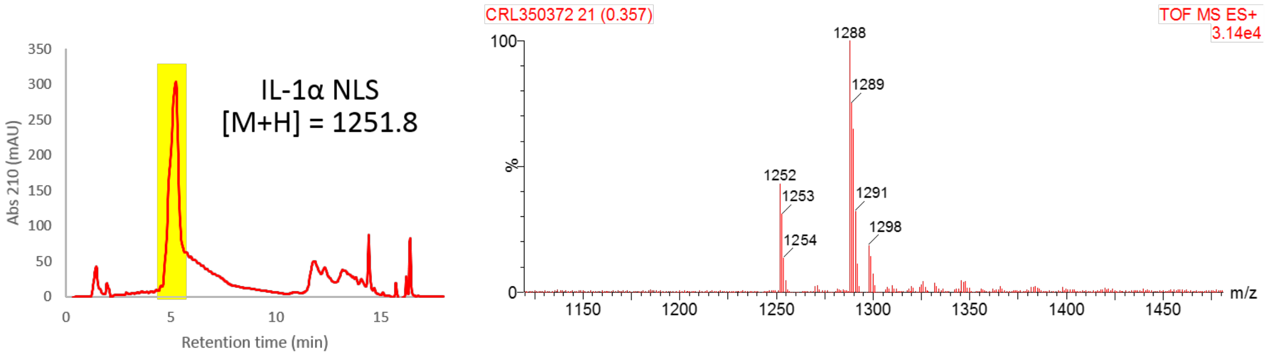

Supplementary Figure 19. HPLC trace and MS spectrum of FmocNH-KVLKKRRC-CONH$_2$ (IL-1α NLS) peptide for attachment to CNTs. Yellow region in designates portion of the sample collected.

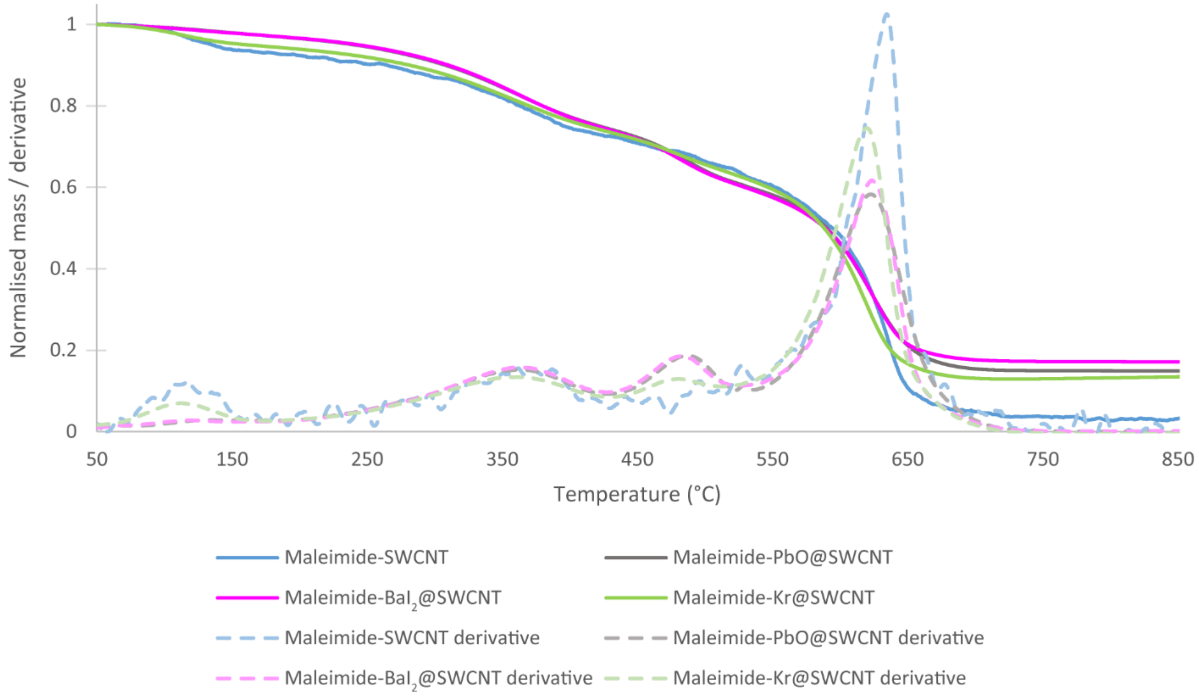

Supplementary Figure 20. TGA curves of maleimide functionalised filled SWCNTs measured in air.

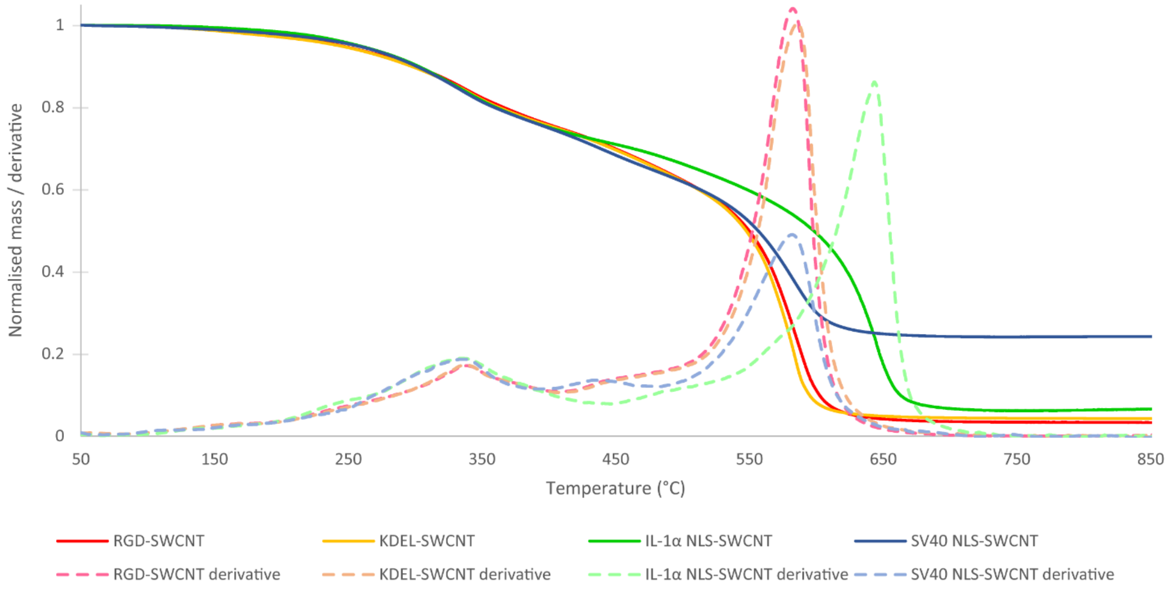

Supplementary Figure 21. TGA curves of peptide decorated empty SWCNTs measured in air.

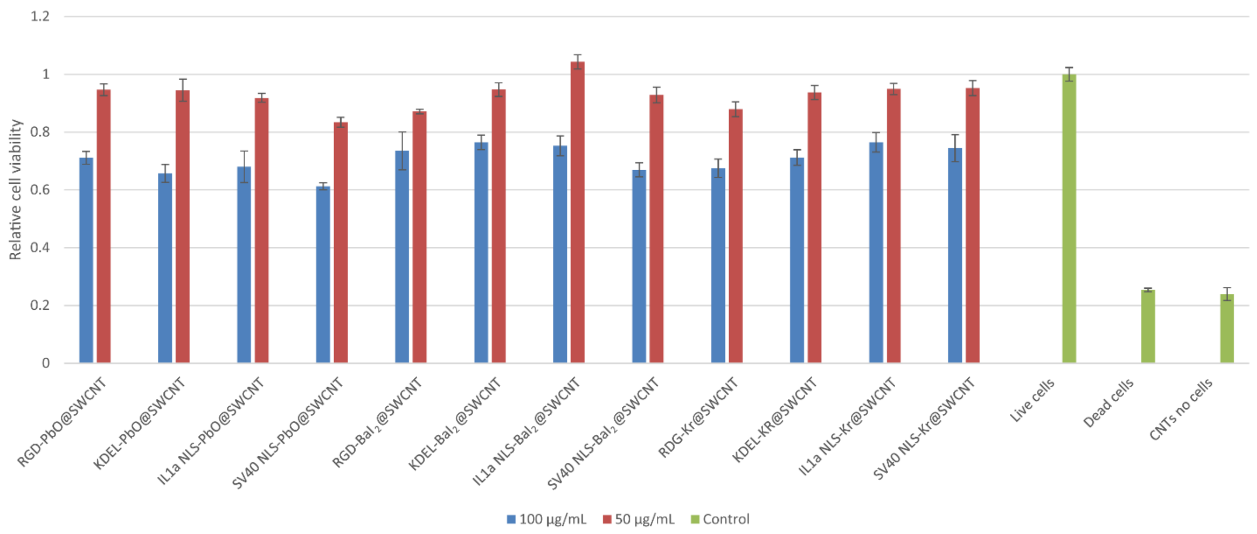

Supplementary Figure 22. MTS assay for filled and decorated SWCNTs administered to HeLa cells. Each sample was measured in triplicate. Heights are mean values, error bars represent the standard deviation.

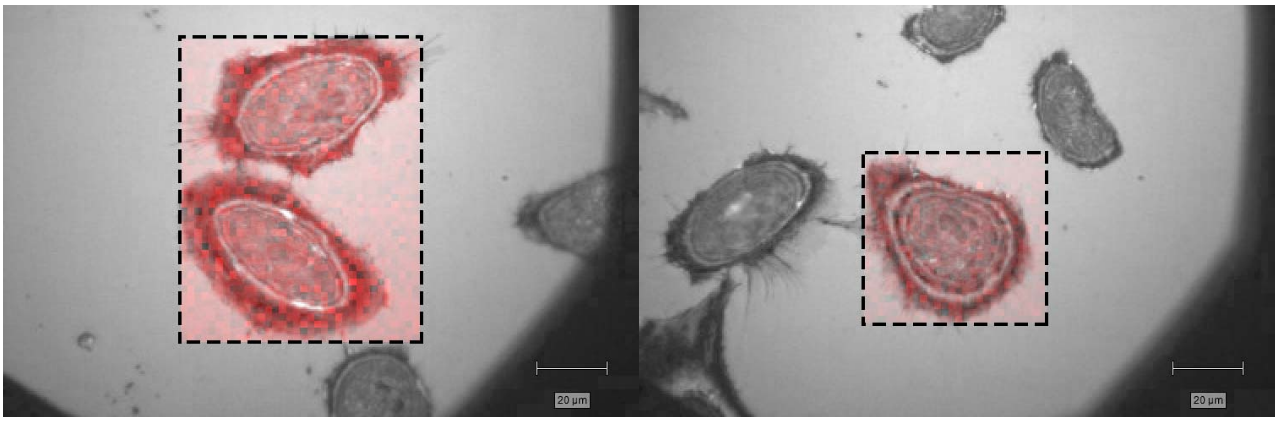

Supplementary Figure 23. Raman G-band (1500 – 1650 cm^{-1}) mapping of untreated fixed HeLa cells. Intensities normalised. Total of five maps collected.

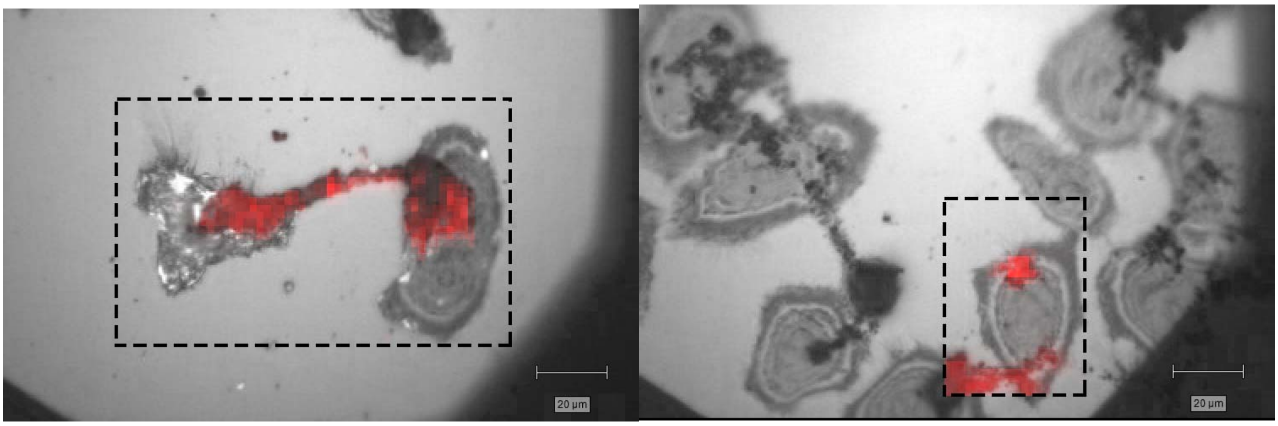

Supplementary Figure 24. Further Raman G-band (1500 – 1650 cm^{-1}) maps of fixed HeLa cells treated with RGD-SWCNTs. Intensities normalised. Total of ten maps collected.

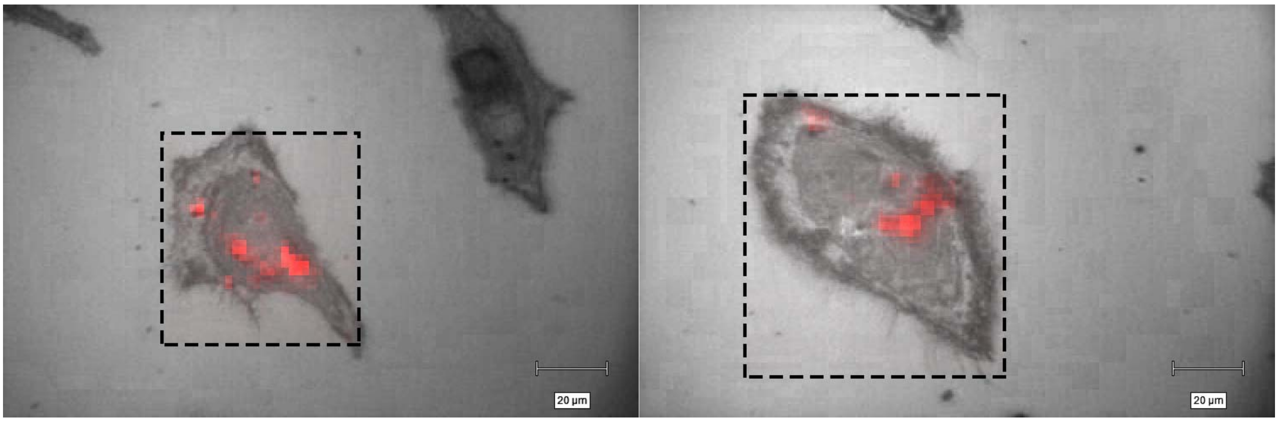

Supplementary Figure 25. Further Raman G-band (1500 – 1650 cm^{-1}) maps of fixed HeLa cells treated with KDEL-SWCNTs. Intensities normalised. Total of nine maps collected.

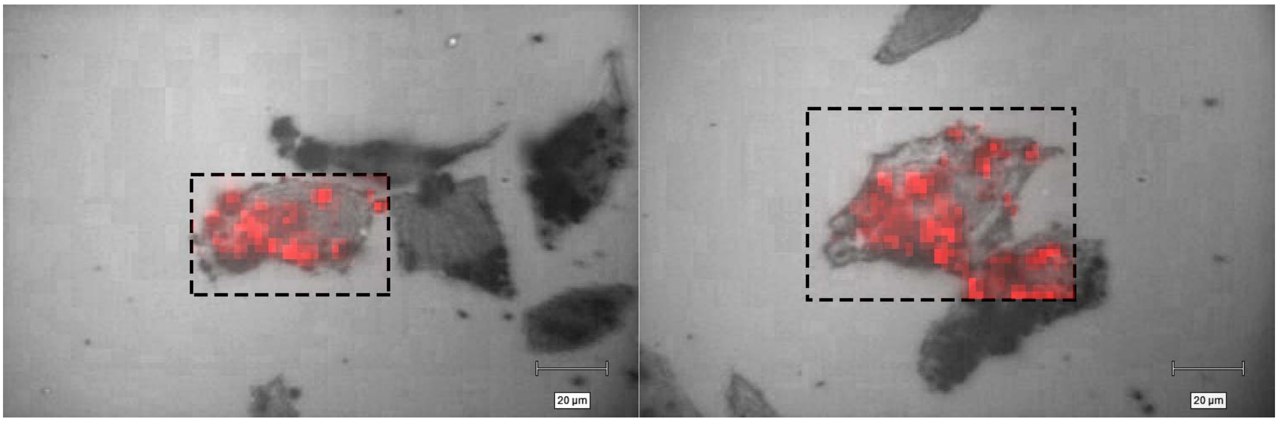

Supplementary Figure 26. Further Raman G-band (1500 – 1650 cm^{-1}) maps of fixed HeLa cells treated with SV40 NLS-SWCNTs. Intensities normalised. Total of nine maps collected.

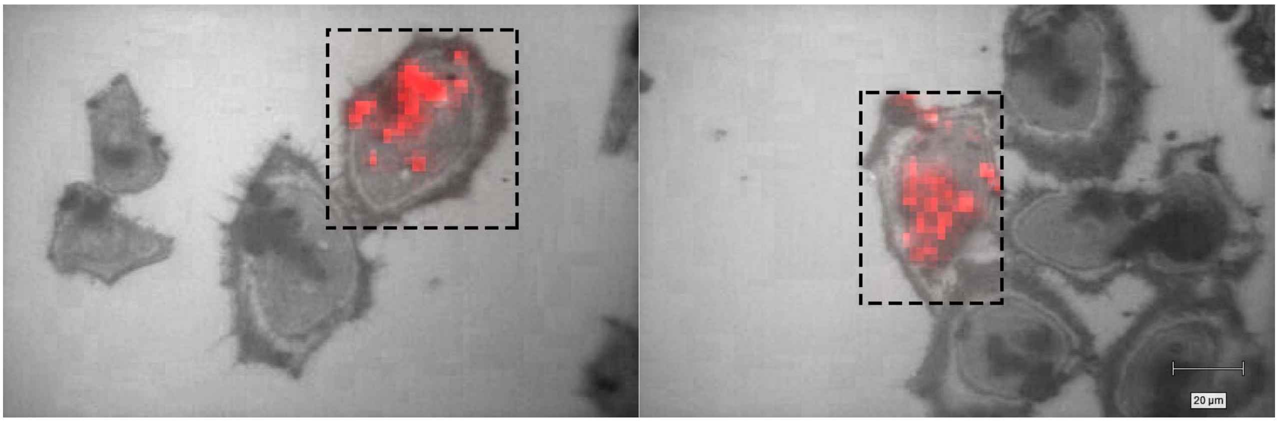

Supplementary Figure 27. Further Raman G-band (1500 – 1650 cm^{-1}) maps of fixed HeLa cells treated with IL-1α NLS-SWCNTs. Total of eleven maps collected.

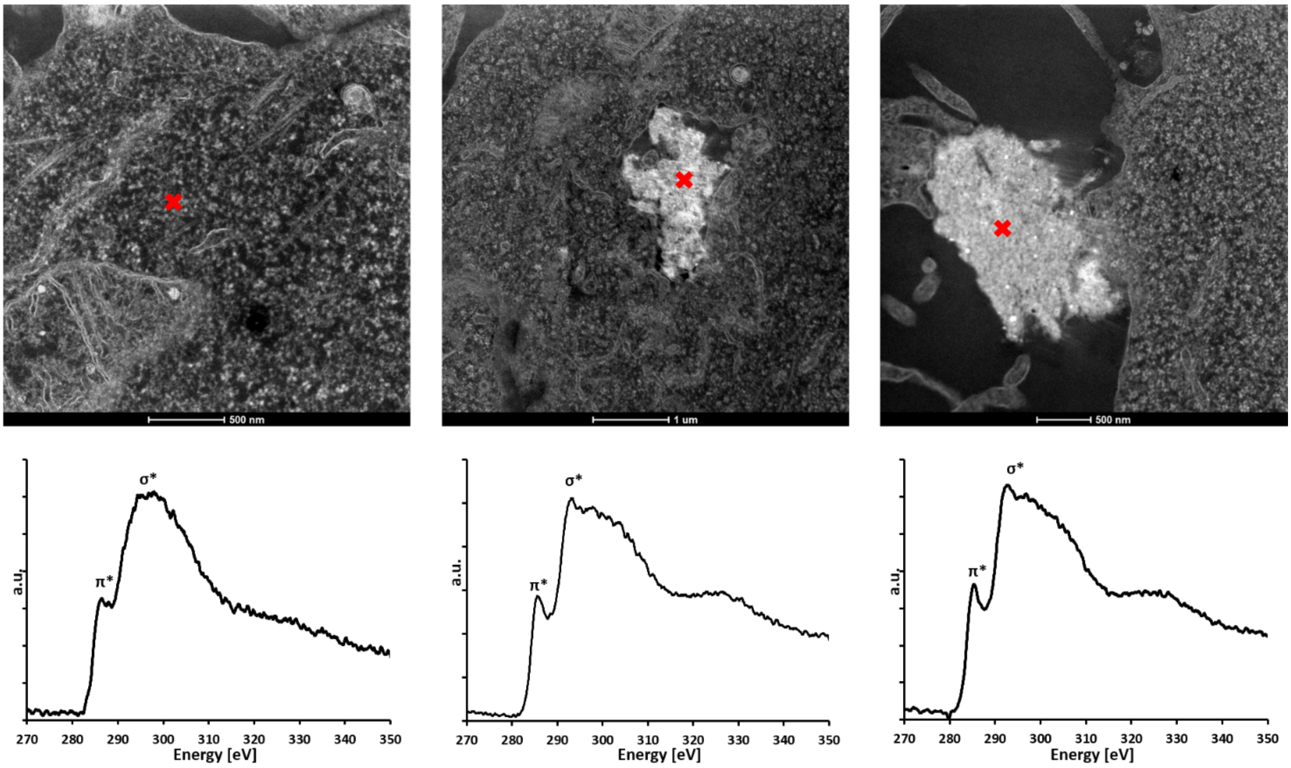

Supplementary Figure 28. Carbon K-edge Electron Energy Loss spectrum (EELS) of the cells (left) SWCNT bundles inside (centre) and outside (right) cells. A peak at 285 eV (1s to π* transition) with a broad band at 290 – 310 eV (σ*) is characteristic of amorphous carbon,[2] while the fine structure of the σ* band, and in particular the sharp peak at 293 eV, is indicative of graphitic carbon.[3] The SWCNT bundles can be visually recognised by their high contrast, fibrous texture, bright spots (iron nanoparticle impurities), and 'smearing' due to resistance to the microtome. Total of seven areas imaged with point spectra collected.

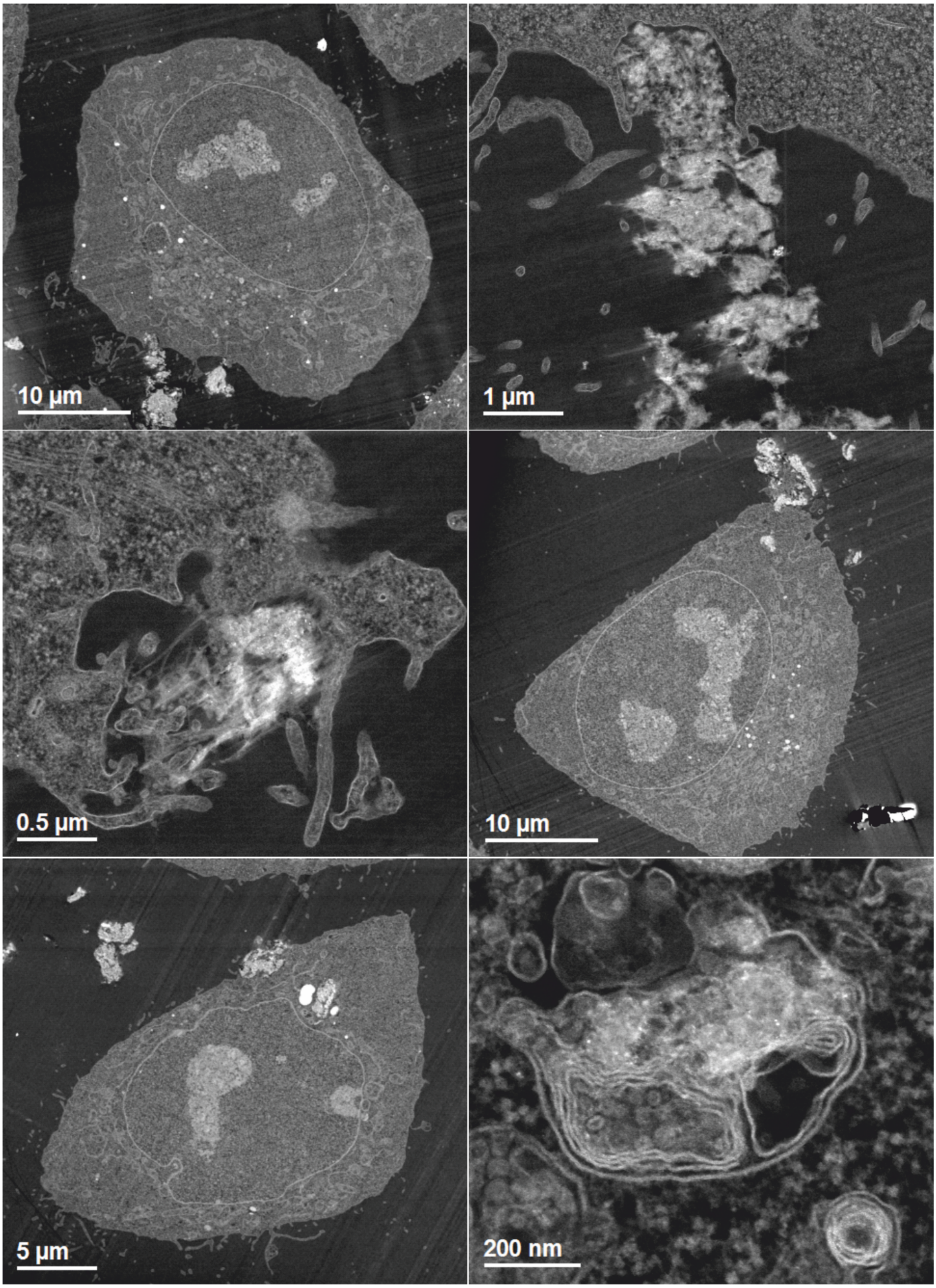

Supplementary Figure 29. Further HAADF-STEM images of HeLa cells treated with IL-1α NLS-SWCNTs. Total of 38 images collected.

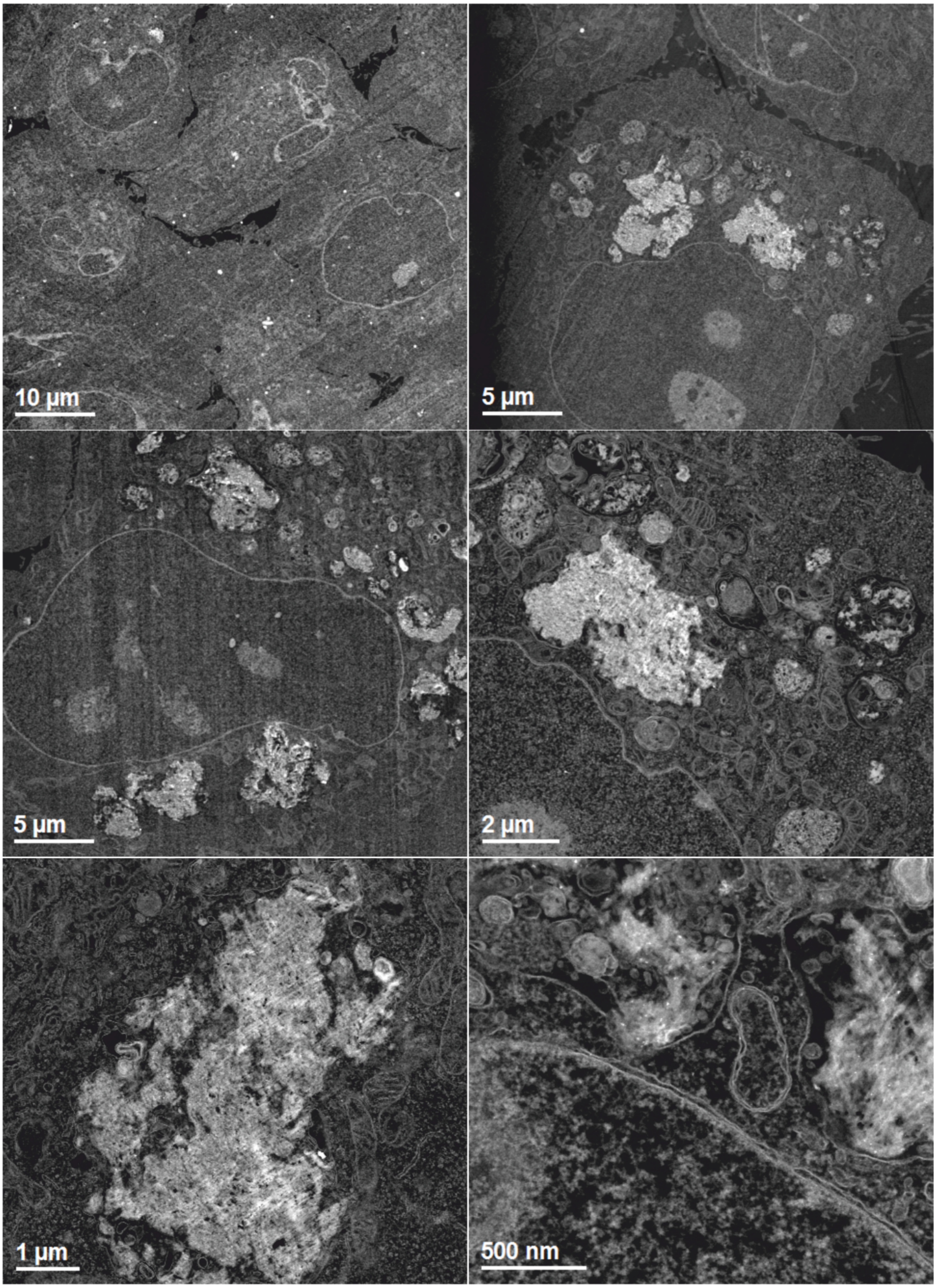

Supplementary Figure 30. Further HAADF-STEM images of HeLa cells treated with SV40 NLS-SWCNTs. Note proximity to nuclear envelope. Total of 41 images collected.

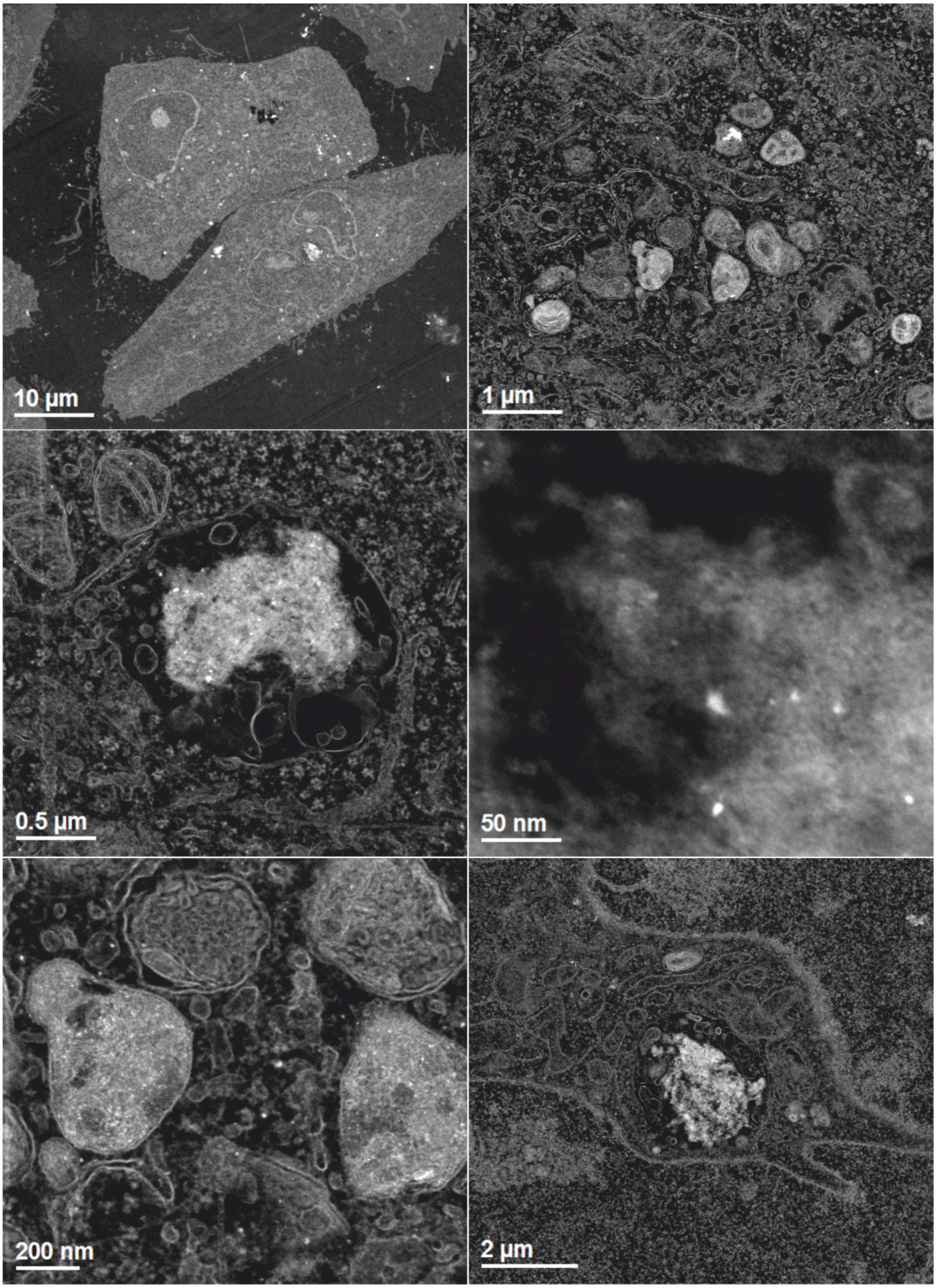

Supplementary Figure 31. Further HAADF-STEM images of HeLa cells treated with KDEL-SWCNTs. Note that rough ER is difficult to distinguish from SWCNT bundles in small cellular compartments. Total of 35 images collected.

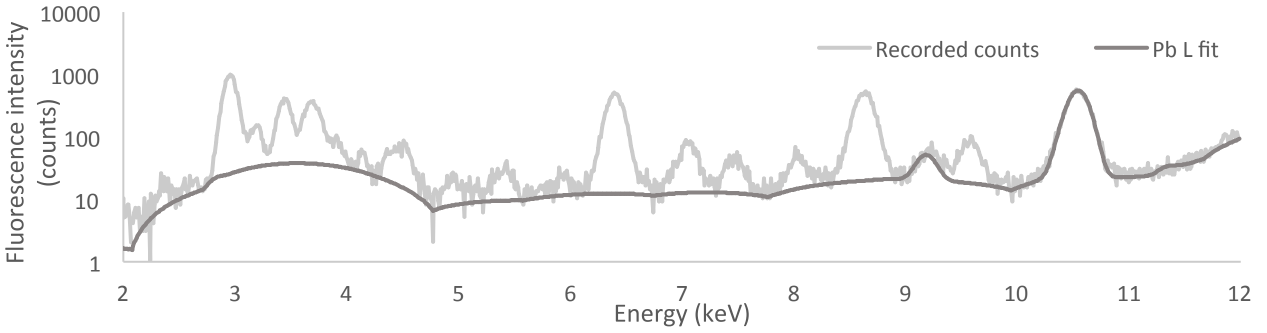

Supplementary Figure 32. Fitting of Pb L signals for the point (62,13) in map shown in Supplementary Figure 41 (RGD targeting).

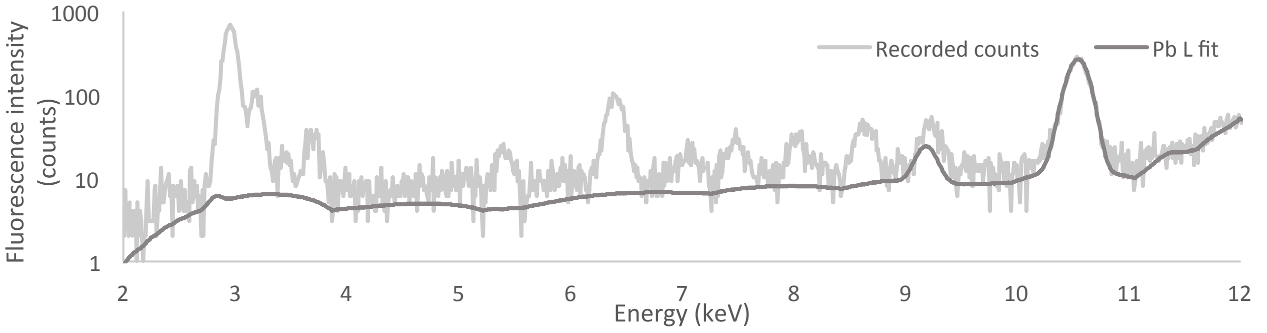

Supplementary Figure 33. Fitting of Pb L signals for the point (58,27) in map shown in Supplementary Figure 43 (IL-1α NLS targeting).

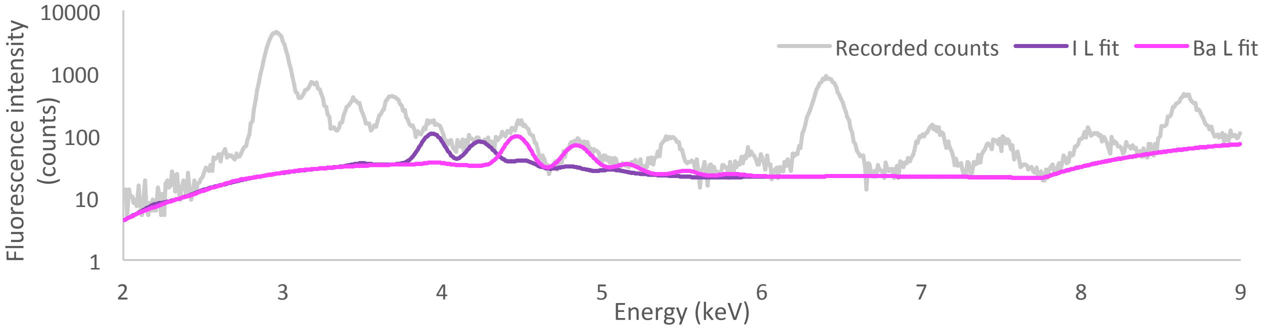

Supplementary Figure 34. Fitting of Ba and I L signals for the point (74,45) in map shown in Supplementary Figure 44 (RGD targeting).

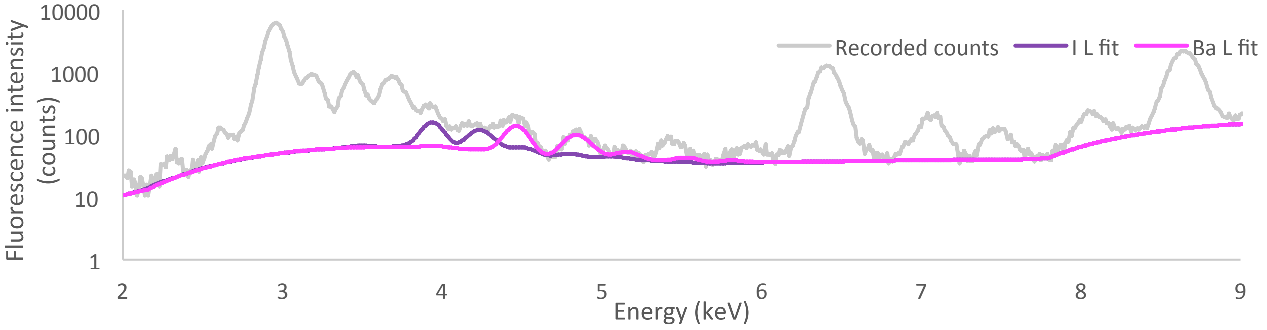

Supplementary Figure 35. Fitting of Ba and I L signals for the point (9,42) in map shown in Supplementary Figure 47 (SV40 targeting).

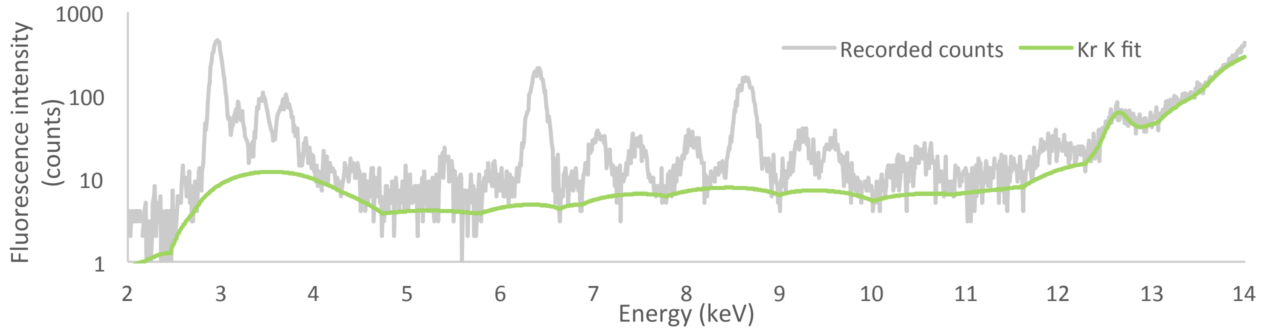

Supplementary Figure 36. Fitting of Kr K signal for the point (5,20) in map shown in Supplementary Figure 48 (RGD targeting).

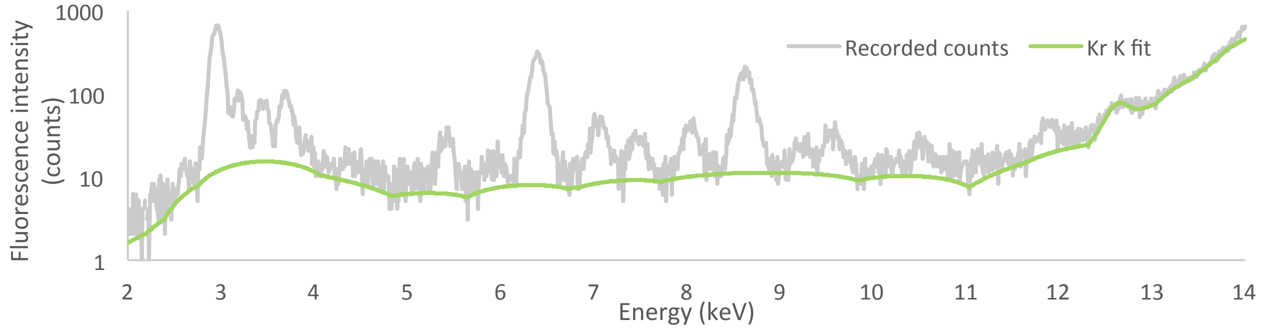

Supplementary Figure 37. Fitting of Kr K signal for the point (24,22) in map shown in Supplementary Figure 51 (SV40 targeting)

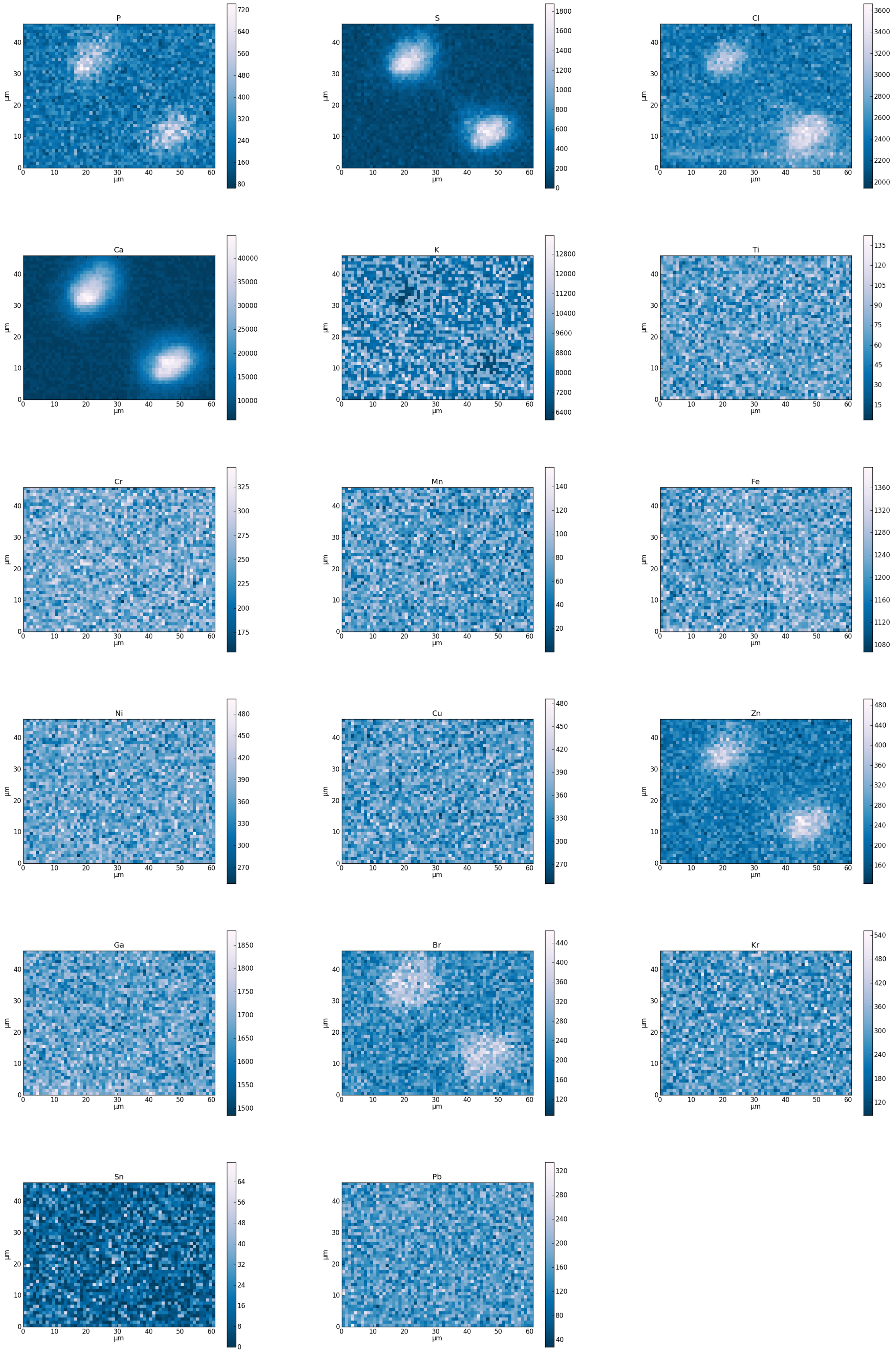

Supplementary Figure 38. XRF map of fixed HeLa cells in the absence of CNTs, scaled linearly. 60 x 45 µm.

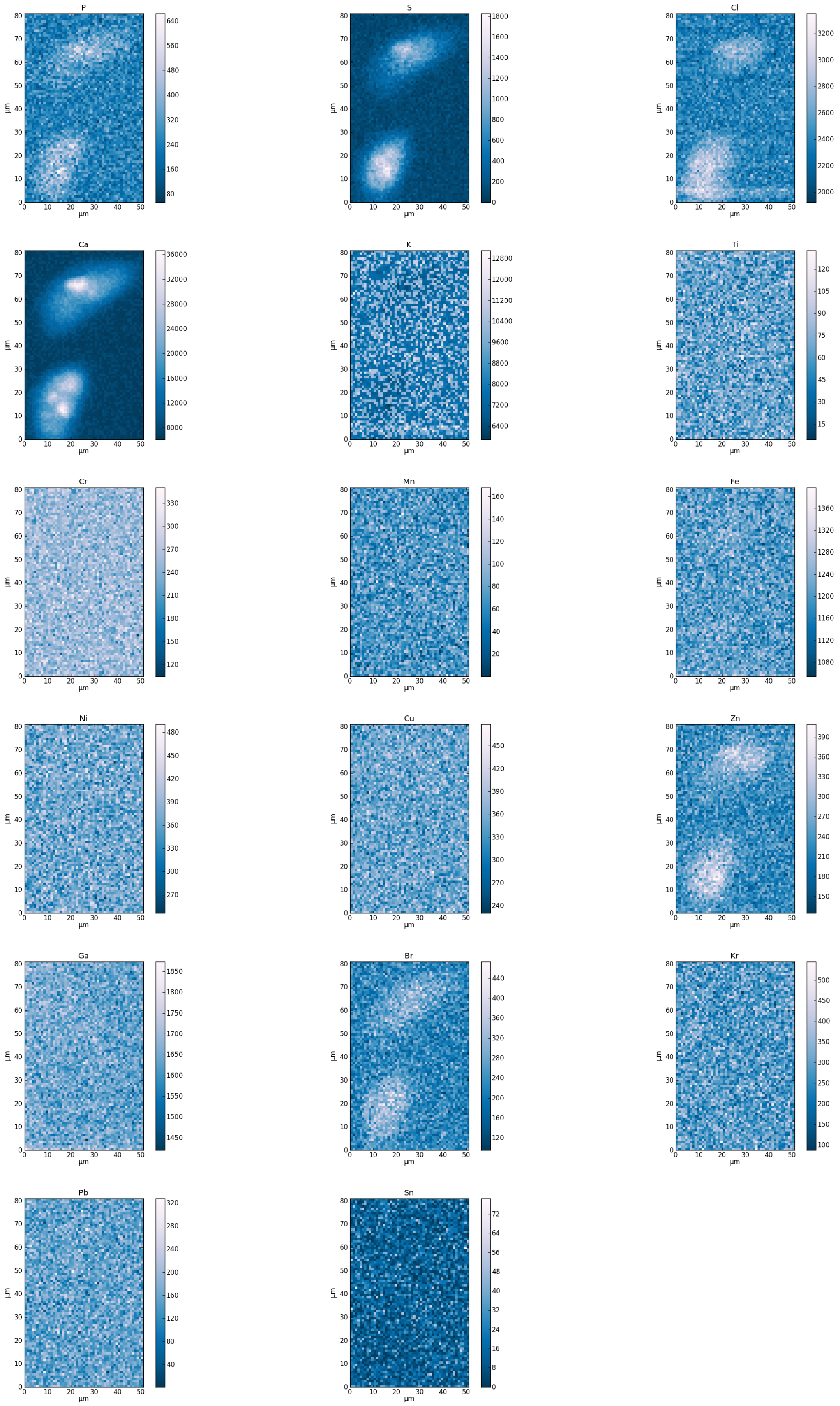

Supplementary Figure 39. XRF map of fixed HeLa cells in the absence of CNTs, scaled linearly. 50 x 80 µm.

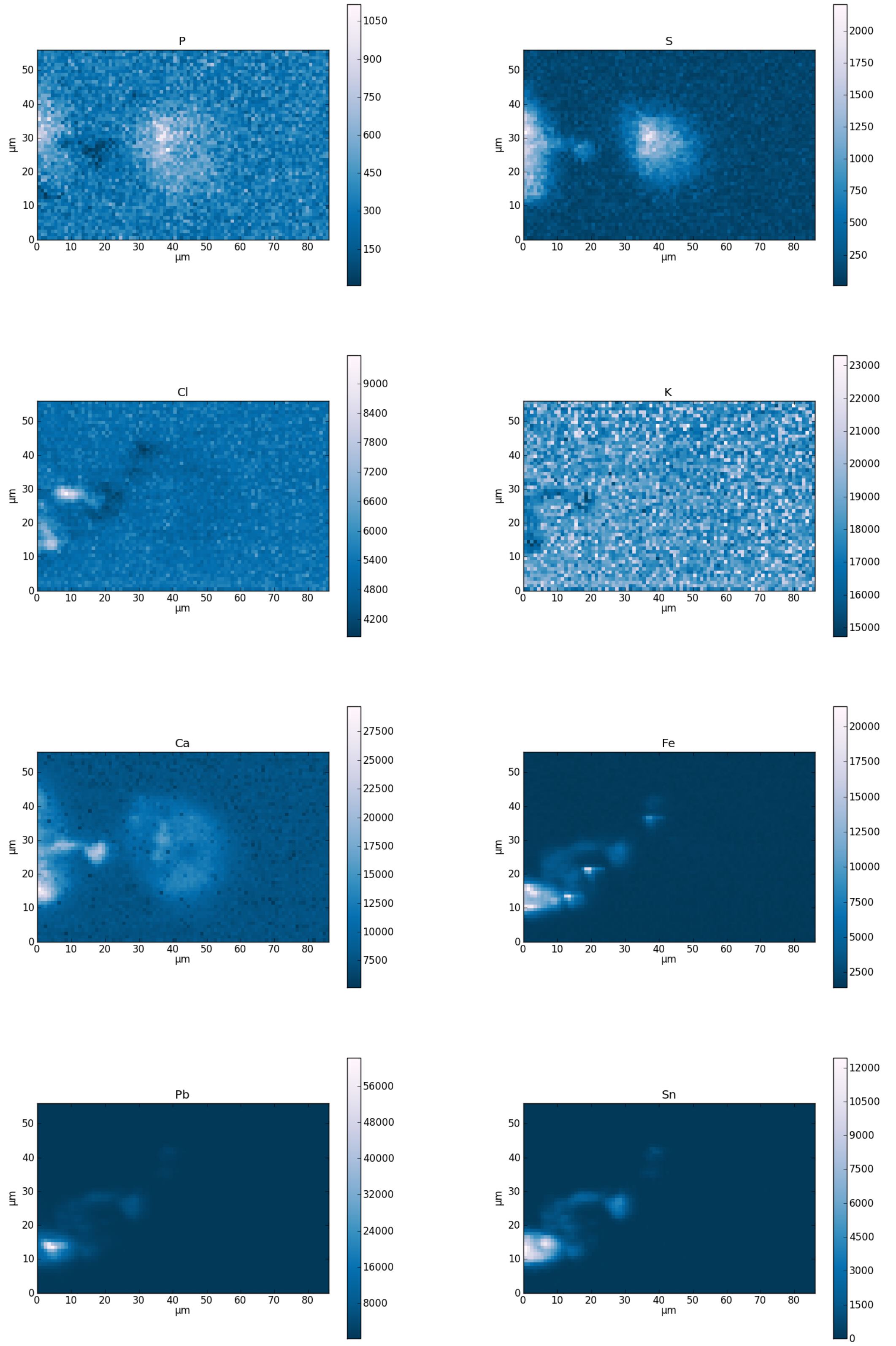

Supplementary Figure 40. XRF map of fixed HeLa cells treated with RGD-decorated PbO@SWCNT, scaled linearly. 85 x 55 µm.

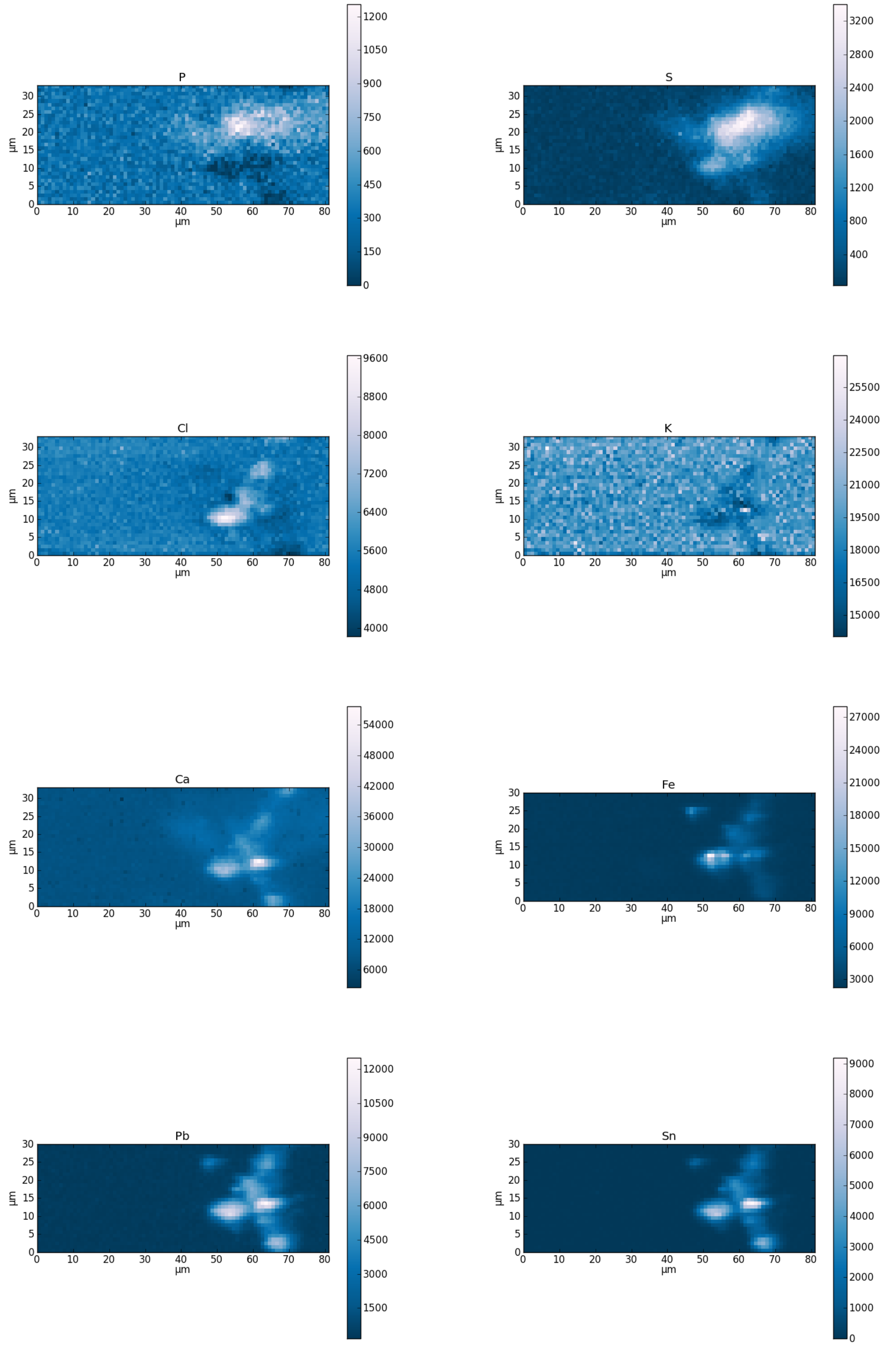

Supplementary Figure 41. XRF map of fixed HeLa cells treated with RGD-decorated PbO@SWCNT, scaled linearly. 80 x 29 µm.

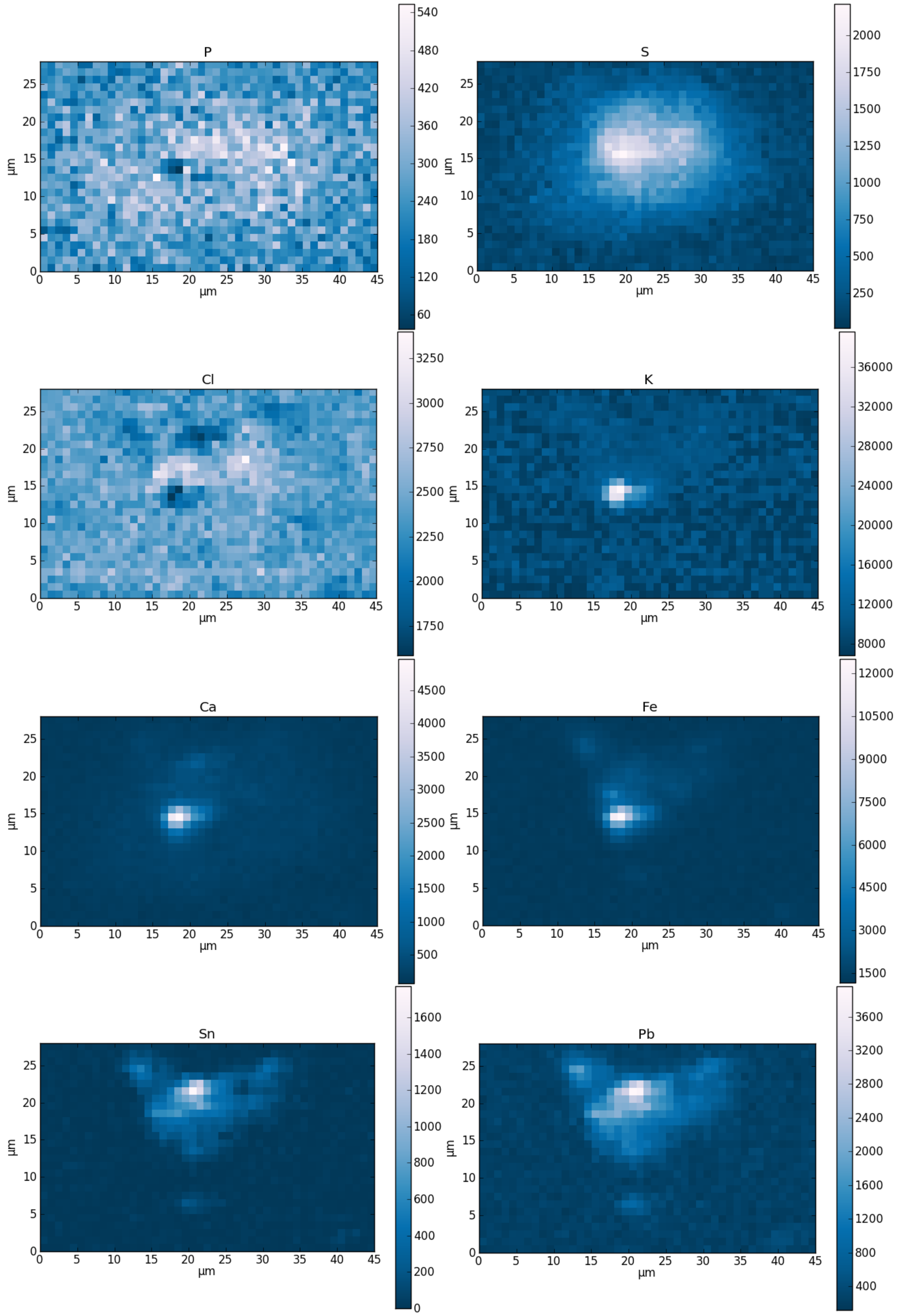

Supplementary Figure 42. XRF map of fixed HeLa cells treated with IL-1α NLS-decorated PbO@SWCNT, scaled linearly. 45 x 28 µm.

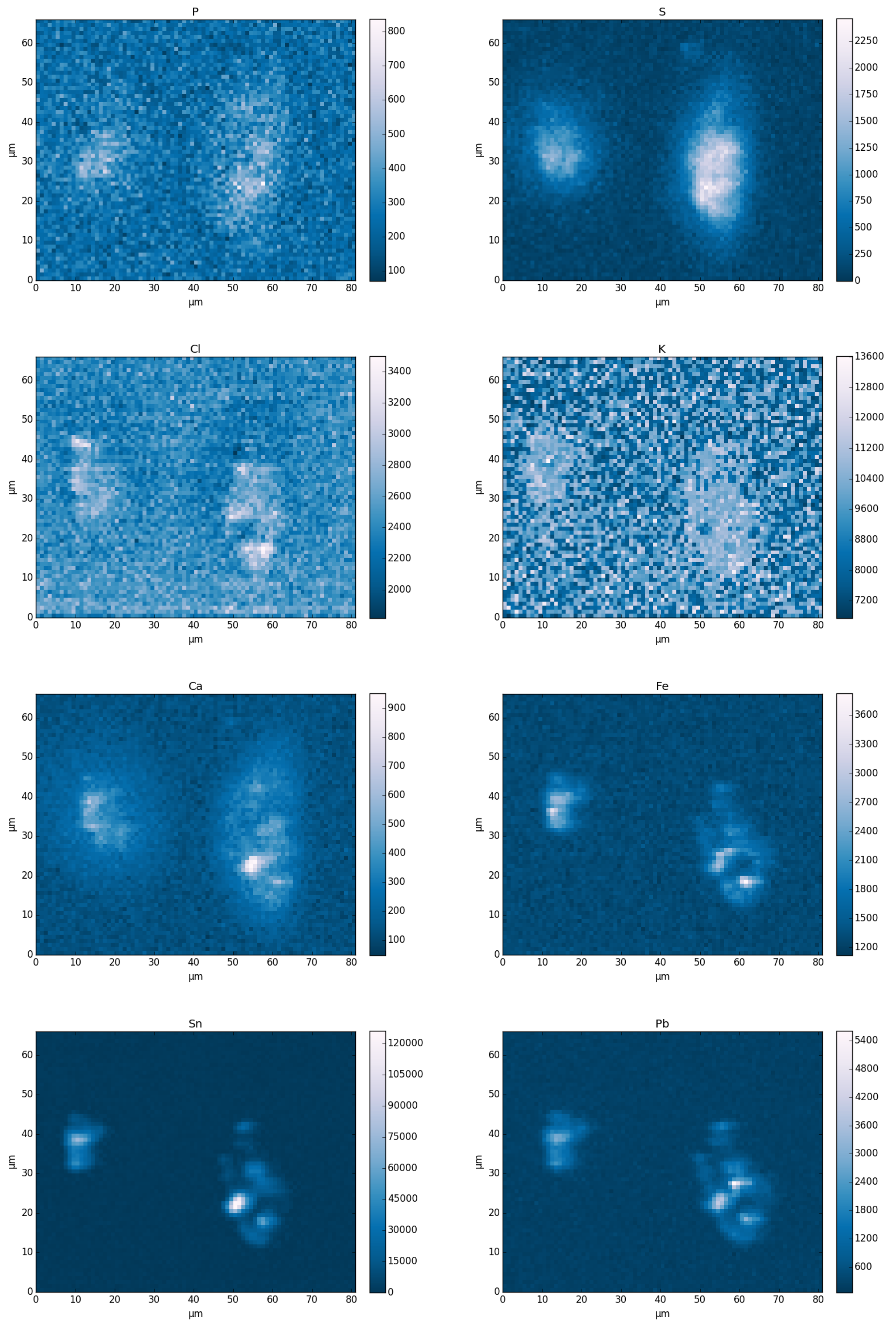

Supplementary Figure 43. XRF map of fixed HeLa cells treated with IL-1α NLS-decorated PbO@SWCNT, scaled linearly. 80 x 65 µm.

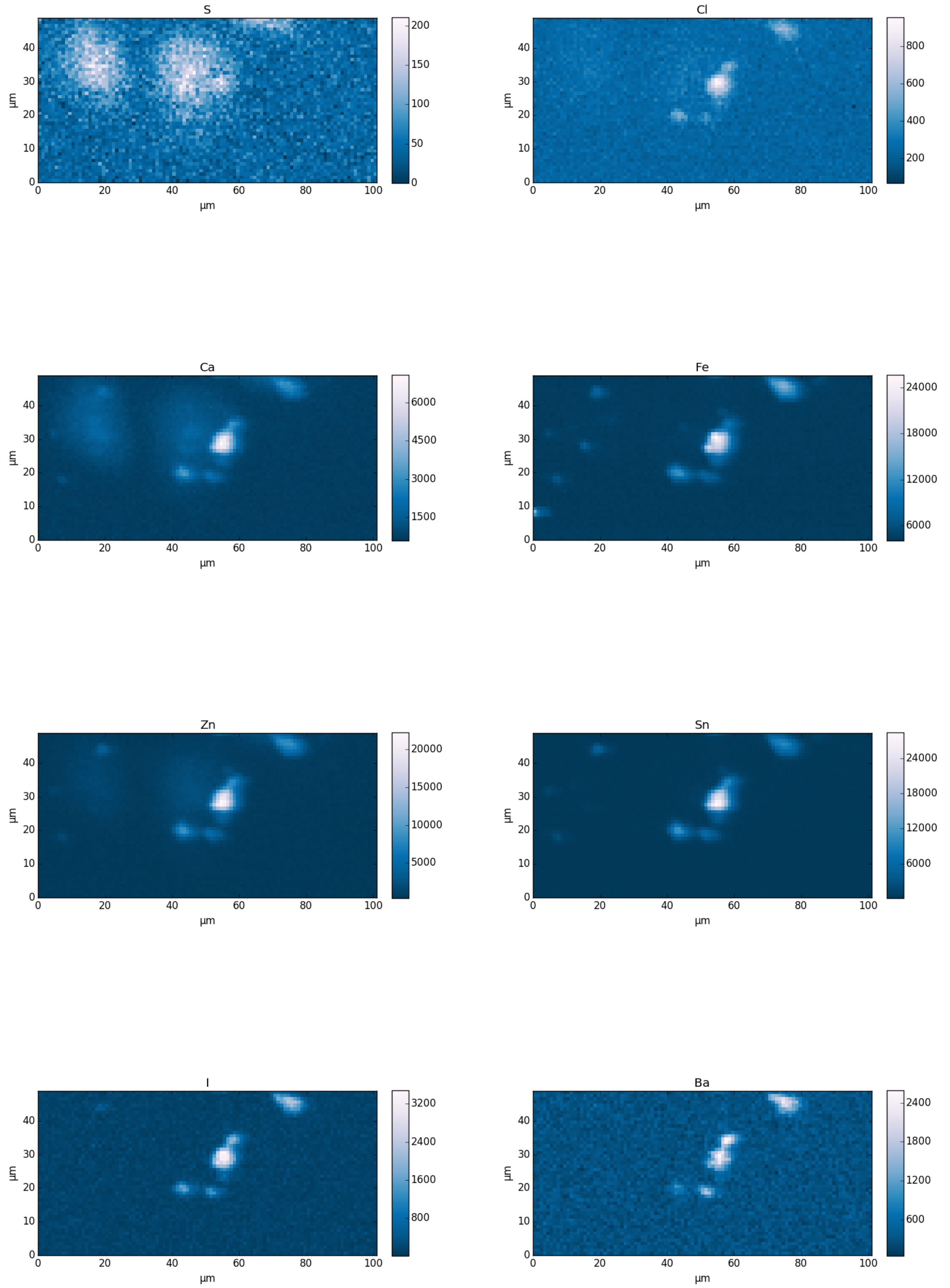

Supplementary Figure 44. XRF map of fixed HeLa cells treated with RGD-decorated BaI_2@SWCNT, scaled linearly. 100 x 48 μm.

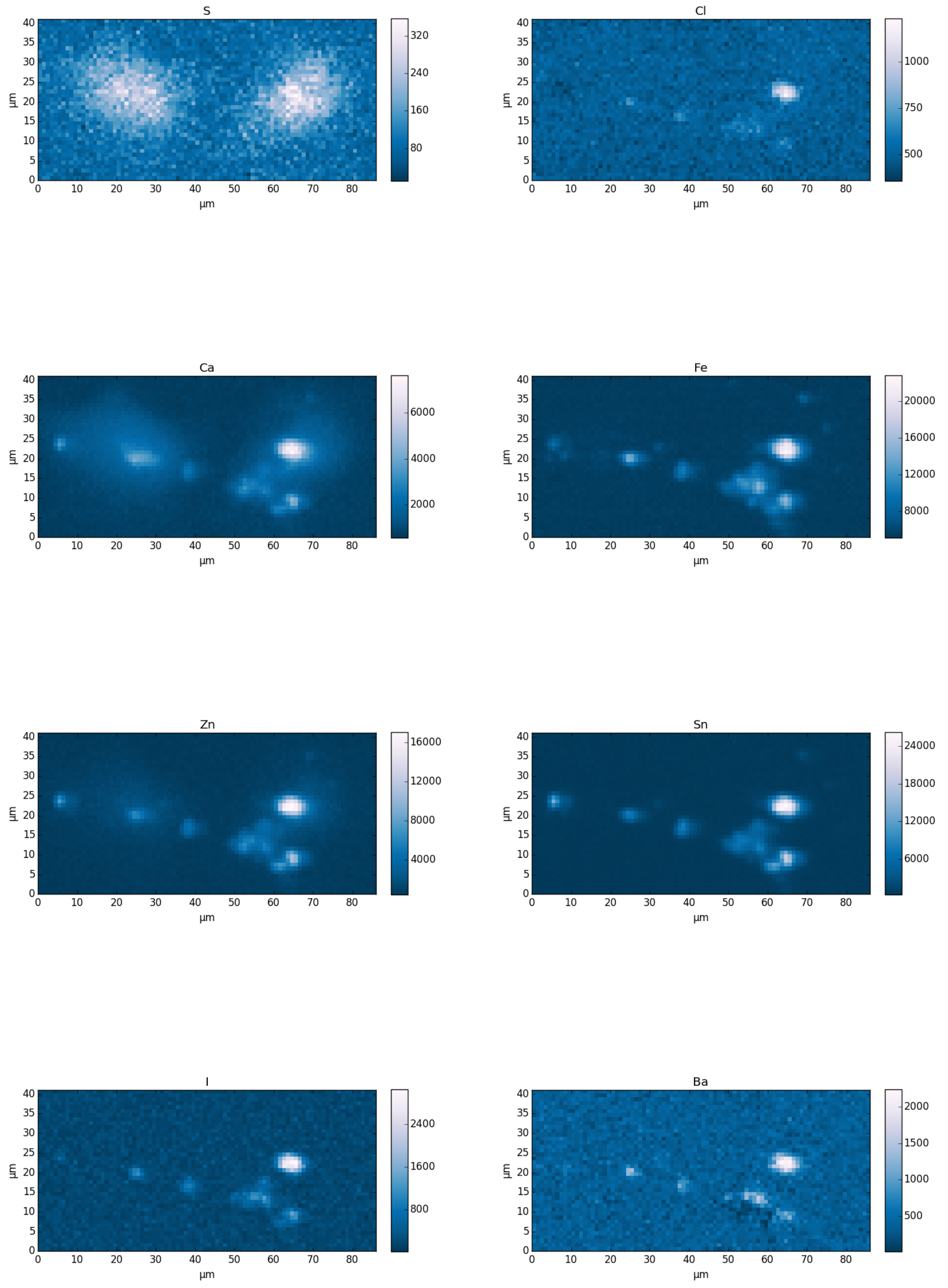

Supplementary Figure 45. XRF map of fixed HeLa cells treated with RGD-decorated BaI_2@SWCNT, scaled linearly. 85 x 40 µm.

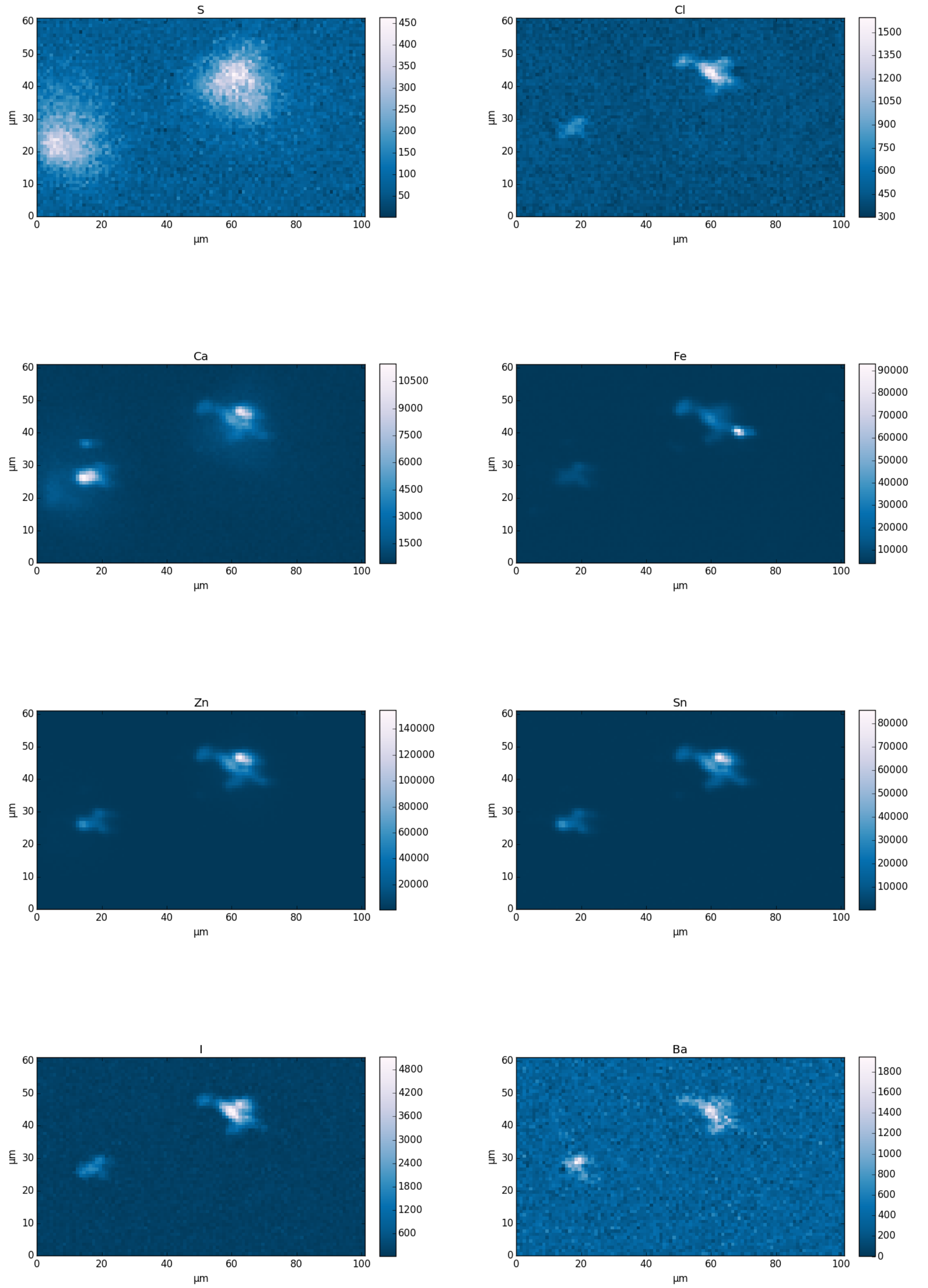

Supplementary Figure 46. XRF map of fixed HeLa cells treated with SV40 NLS-decorated BaI_2@SWCNT, scaled linearly. 100 x 59 μm.

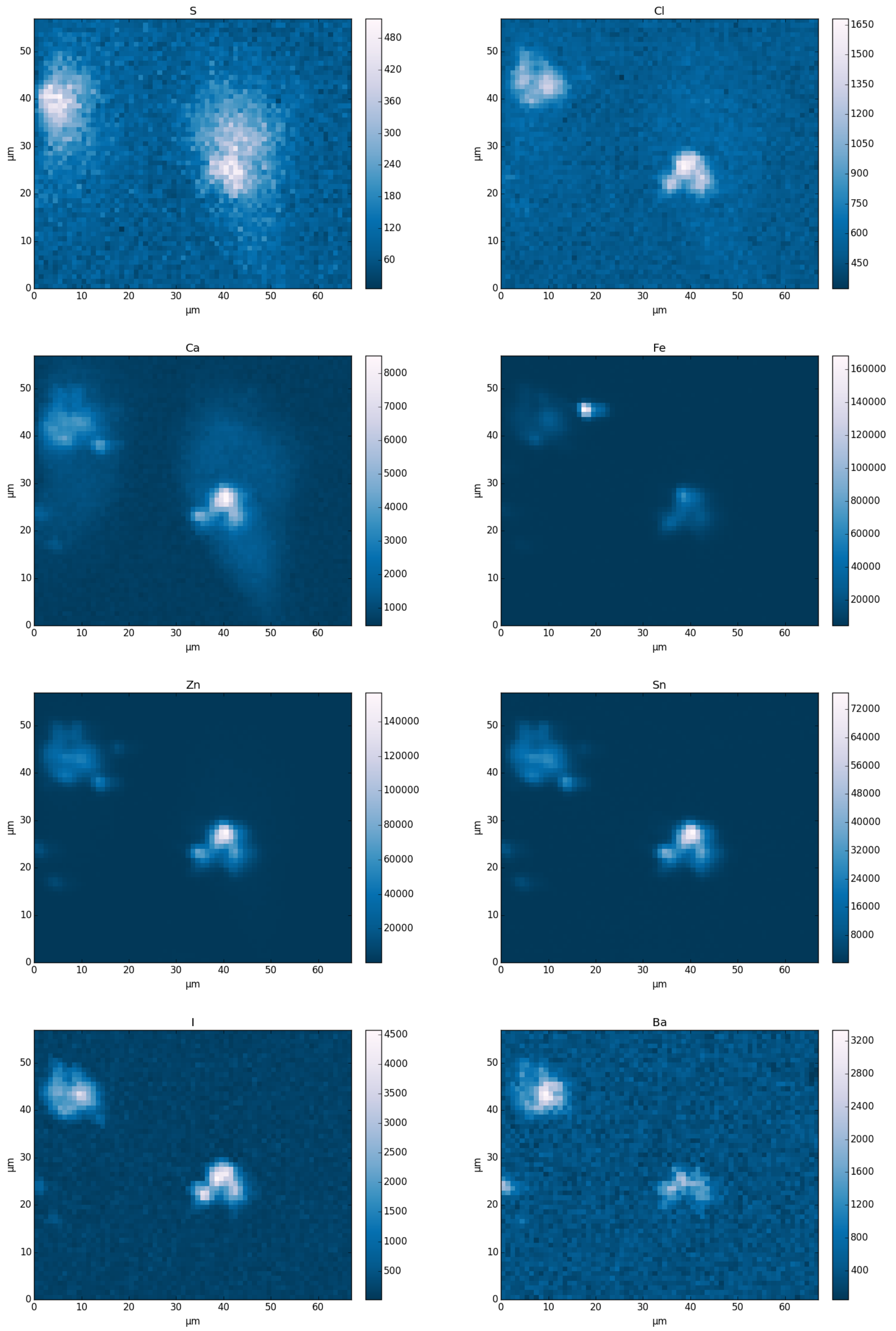

Supplementary Figure 47. XRF map of fixed HeLa cells treated with SV40 NLS-decorated BaI_2@SWCNT, scaled linearly. 66 x 56 µm.

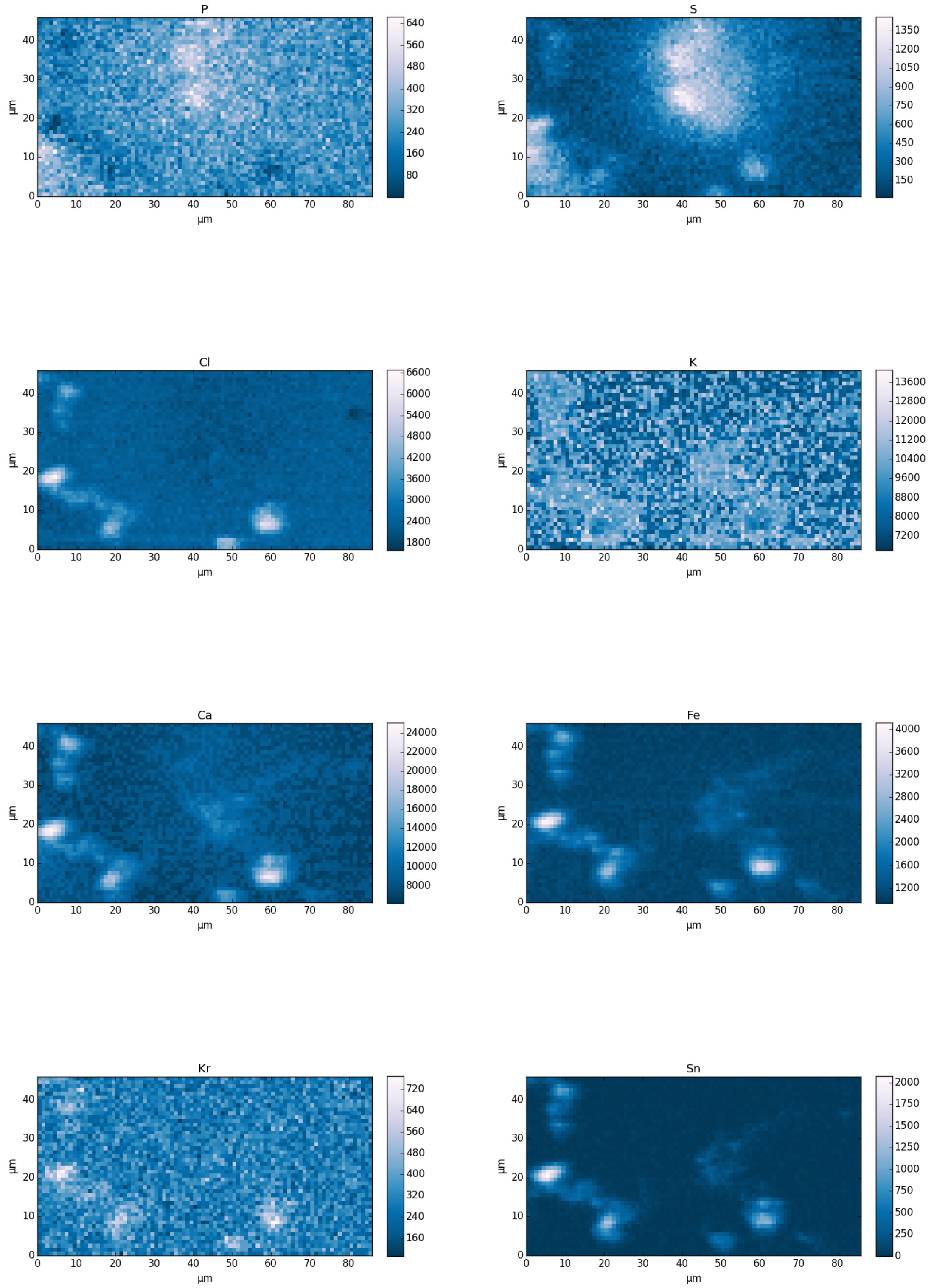

Supplementary Figure 48. XRF map of fixed HeLa cells treated with RGD-decorated Kr@SWCNT, scaled linearly. 80 x 45 µm.

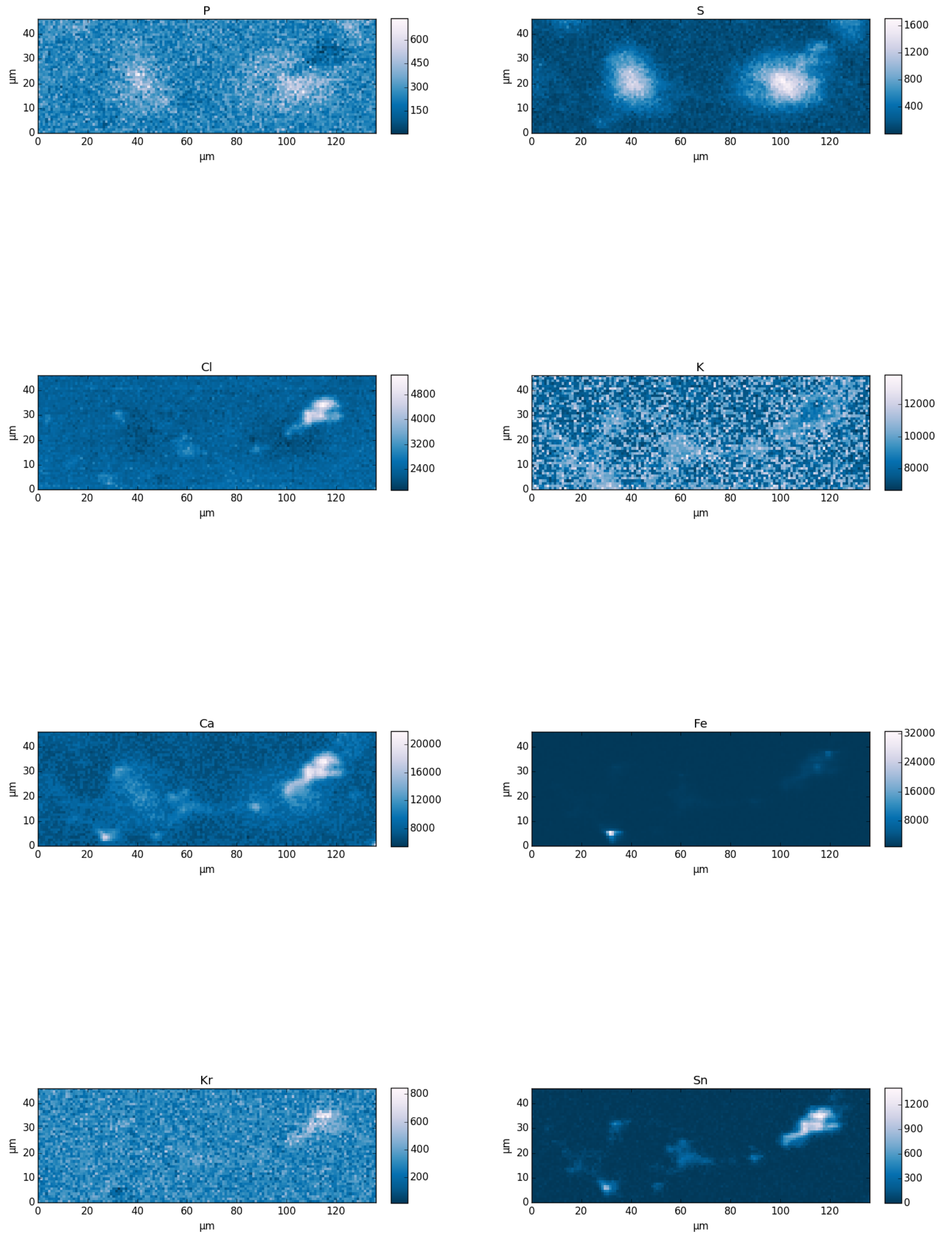

Supplementary Figure 49. XRF map of fixed HeLa cells treated with RGD-decorated Kr@SWCNT, scaled linearly. 135 x 45 µm.

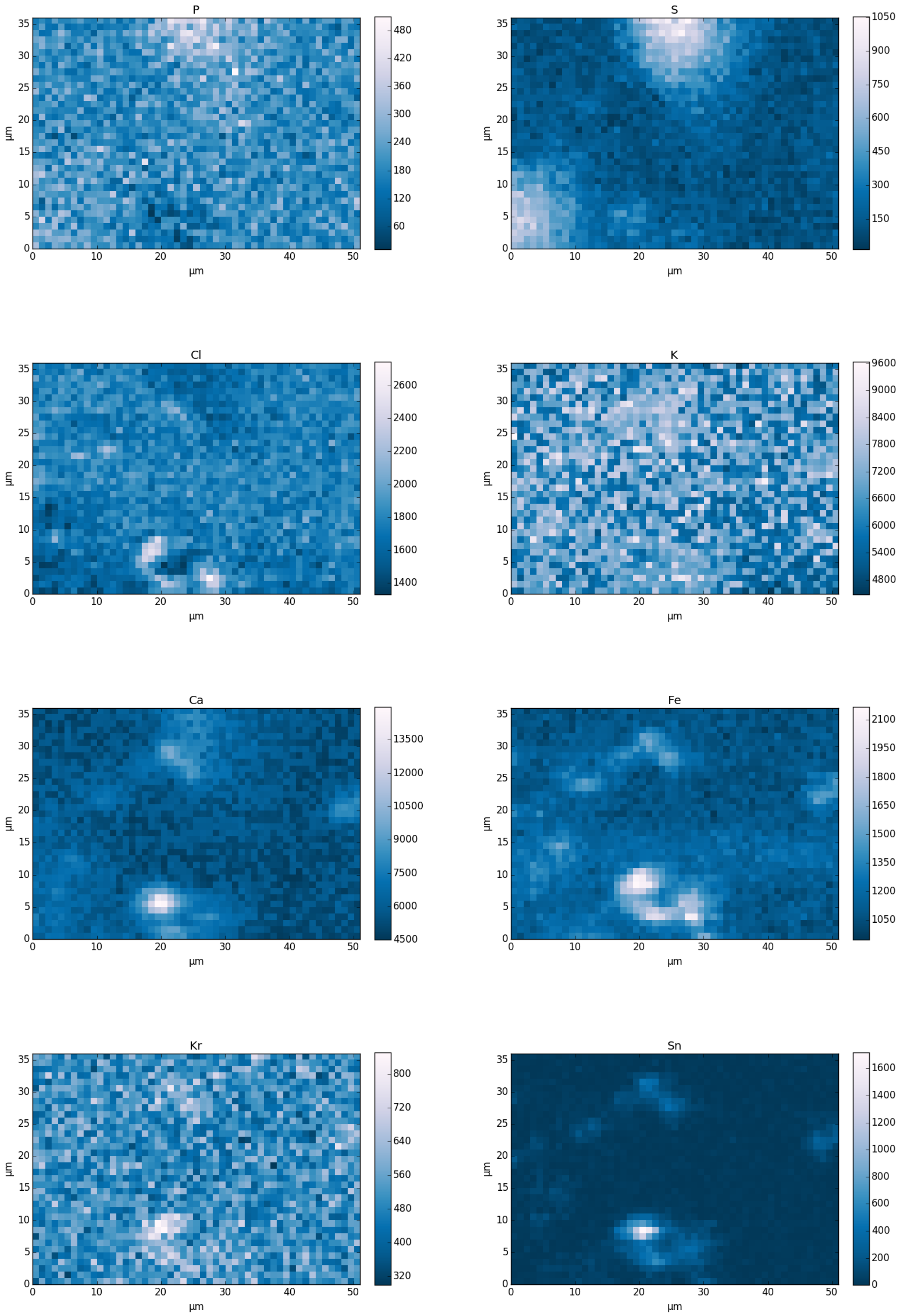

Supplementary Figure 50. XRF map of fixed HeLa cells treated with RGD-decorated Kr@SWCNT, scaled linearly. 51 x 36 µm.

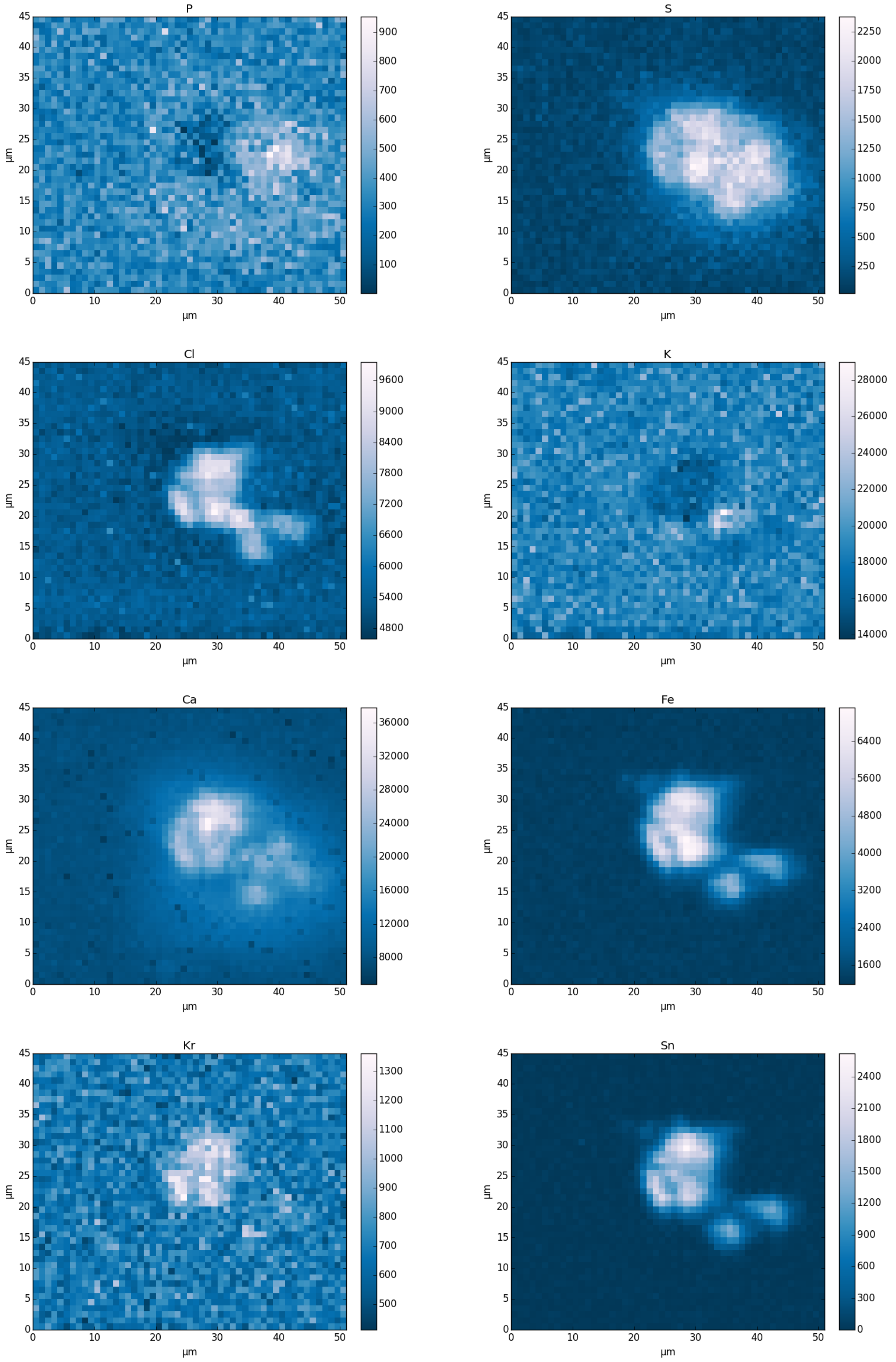

Supplementary Figure 51. XRF map of fixed HeLa cells treated with SV40 NLS-decorated Kr@SWCNT, scaled linearly. 50 x 44 µm.

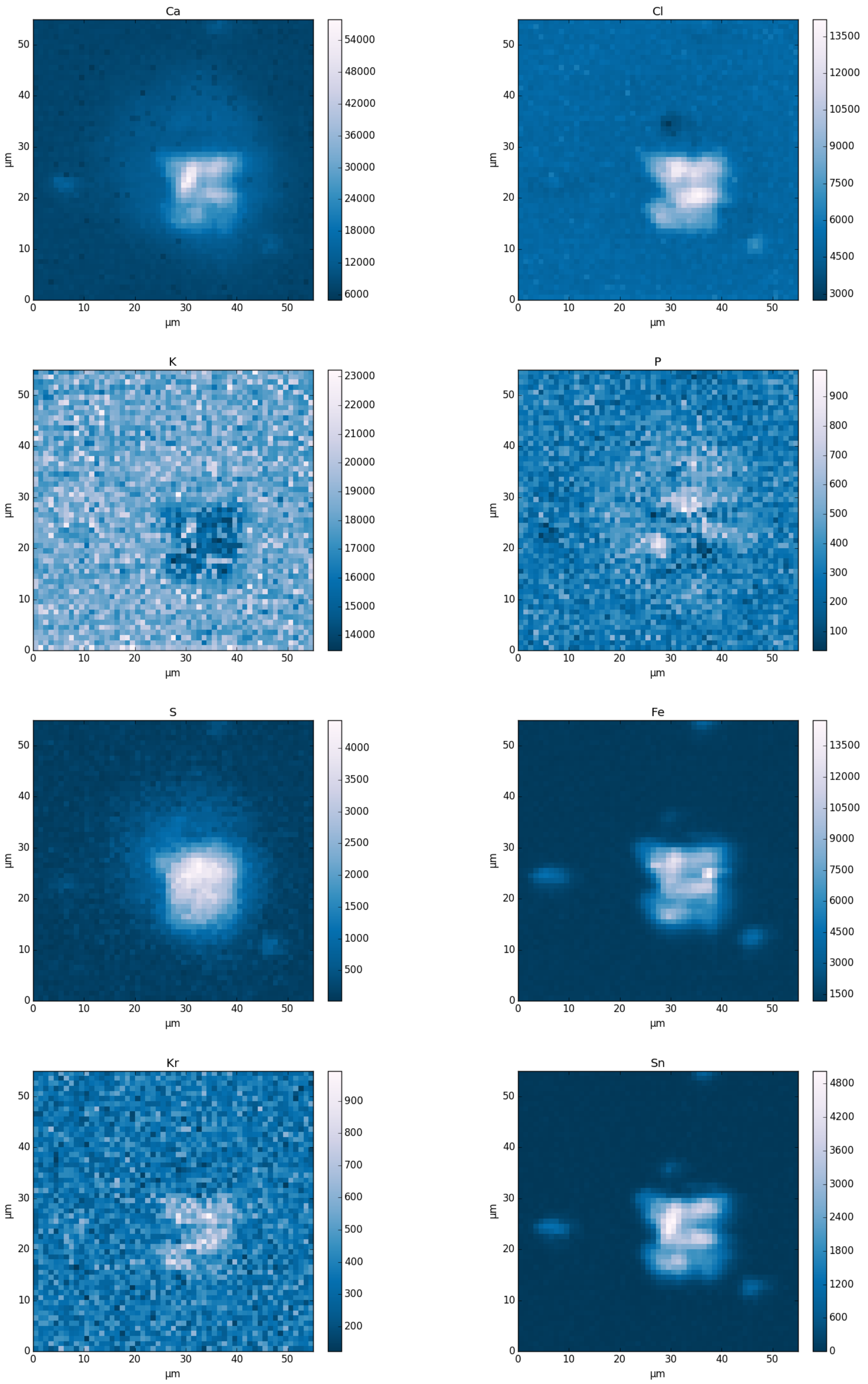

Supplementary Figure 52. XRF map of fixed HeLa cells treated with SV40 NLS-decorated Kr@SWCNT, scaled linearly. 54 x 54 µm.

Supplementary Tables

Supplementary Table 1. ICP-MS analysis of supernatants from washed of filled SWCNTs at 1 mg mL^{-1}.

Sample	Metal present (ug mL^{-1}, corrected for dilution)
PbO@SWCNT (1st HCl wash)	71.820 Pb
PbO@SWCNT (2nd HCl wash)	4.5849 Pb
PbO@SWCNT (water wash)	39.889 Pb
BaI_2@SWCNT (water wash)	0.2486 Ba

Supplementary Table 2. Decoration yields for empty SWCNTs as determined by TGA and Fmoc numbering.

Decoration	Conjugation yield (Fmoc) /µmol mg^{-1}	Conjugation yield (TGA) /µmol mg^{-1}
Maleimide	n/a	1.01
RGD	0.81	0.37
KDEL	0.62	0.30
IL-1α NLS	0.27	0.21
SV40 NLS	0.24	0.22

Supplementary Table 3. Yields (mg) for attachment of peptides to filled SWCNTs starting from 2.0 mg of maleimide-decorated filled SWCNT.

Filling	RGD	KDEL	IL-1α NLS	SV40 NLS
**Kr**	1.17	0.43	0.55	0.70
**BaI_2**	1.16	1.23	1.17	1.47
**PbO**	0.63	0.71	1.08	0.68

Supplementary Table 4. Conjugation yields (µmol mg^{-1}) for attachment of peptides to filled SWCNTs determined by Fmoc numbering.

Filling	RGD	KDEL	IL-1α NLS	SV40 NLS
**Kr**	1.58	1.09	1.36	0.46
**BaI_2**	1.18	0.45	0.28	0.29
**PbO**	1.92	1.13	0.48	0.77

Supplementary Table 5. XRF mapping parameters

**Beam size**	2.0 x 2.3 µm
**Pixel increment**	1 µm
**Exposure time per pixel**	1 sec
**Photon flux density**	~10^{11} ph $µm^{-2}$ s^{-1} (5-10 keV), ~5 × 10^{10} ph $µm^{-2}$ s^{-1} (14 keV)
**Detector type**	6-element silicon drift detector (SGX)
**Detector distance**	70 mm
**Detector area**	530 mm^2
**Solid angle**	~0.1 sr

Supplementary Discussion

Although the fillings are toxic to humans and animals, it is important to note that their effect on cells grown *in vitro* cannot be expected to follow the same trends: Ba is a neurotoxin, while Pb affects a number of organs (such as heart, bones, intestines, and kidneys). Nonetheless, for reference, the lethal oral dose of Pb for humans is ca. 450 mg kg^{-1} of subject,[4] and that of Ba ca. 50 mg kg^{-1}.[5] The exposure inhalation limits considered immediately dangerous to life or health (IDLH) are 0.10 µg mL^{-1} of atmosphere and 0.25 µg mL^{-1} respectively. Our experiments dosed cells with 0.14 (Pb) and 0.031 (Ba) mg kg^{-1} of cells, which is well under to lethal oral dose, but 14 (Pb) and 3.1 (Ba) µg mL^{-1} of media which is much higher than the IDLH values.

Supplementary Methods

Assessment of SWCNT end sealing. The sealing efficacy of the synthetic protocol was experimentally assessed in more detail for PbO@SWNT and BaI_2@SWCNT. The existence of Kr@SWCNT at ambient temperature without sealing being theoretically impossible; even more so after high vacuum treatment and given intervals of months between synthesis and measurement necessitated by beamtime allocations.

PbO@SWCNTs. To test the ultimate strength of encapsulation of PbO, PbO@SWCNT (1 mg) was sonicated in 1 mL of concentrated HCl for one hour at ambient temperature. The nanomaterials were separated from the supernatant by centrifugation at 21.100 xg for 10 minutes. The supernatant was diluted 1000x with Mili-Q H_2O and analysed quantitatively by ICP-MS, giving a value of 72 µg mL^{-1} of Pb (Supplementary Table 1) corrected for dilution). This represents 51 % of the total mass of lead in the sample (140 µg, taken from TGA result). A second wash was performed similarly, and analysed, giving a value of just 4.6 µg mL^{-1}. This indicates that approximately half of the PbO was completely sealed within the tubes. Such a result is unexpected, given that all reports to date of the vacuum filling of CNTs which examine the tube ends have found them closed. In this case, the lack of complete closure may relate to the filling materials itself, since previous studies on end-closing of SWCNTs after molten phase filling, typically in the range 700-900 °C, have focused on metal halides. Heating PbO with carbon is a traditional method for the generation of metallic lead, and indeed some metallic particles were observed in the crude mixture here; the oxidising nature of PbO may therefore impede the complete closure of the SWCNTs. Such an effect would not need to affect many sites in order to create holes through which PbO could escape under these conditions. The original report of PbO@SWCNT did not discuss closure of the ends.[6] We note that remaining 44% was not given up in the second wash, and was therefore irreversibly sealed. To test the loss of Pb under more biologically relevant conditions, the same sonication-centrifugation-dilution procedure was conducted using Mili-Q water rather than conc. HCl. A value of 40 µg mL^{-1} of Pb was obtained, representing 28 % of the initially encapsulated lead. It is therefore theoretically possible that this lead could still escape during tissue culture. However, since water sonication and wash steps were performed during each stage of functionalisation of PbO@SWCNT (attachment of maleimide, conjugation of peptide, ring-opening of succinimide, Fmoc deprotection – see below for further details), it is unlikely that any bioavailable, unsealed Pb remains at the time of interaction with cells. This hypothesis was confirmed both by cytotoxicity measurements and XRF maps (particularly the co-location of Pb and CNT-associated Fe).

BaI_2@SWCNTs. Being soluble in water, BaI_2@SWCNT (1 mg) was sonicated in 1 mL of Mili-Q water for one hour at ambient temperature. The nanomaterials were separated from the supernatant by centrifugation at 21.100xg for 10 minutes. The supernatant was diluted 1000x with Mili-Q H_2O and analysed quantitatively by ICP-MS, giving an undiluted value of 0.25 µg mL^{-1} of Pb (Supplementary Table 1). This represents 0.8 % of the total mass of lead in the sample (31 µg, taken from TGA result), and is regarded as negligible. Encapsulation is therefore irreversible; the SWCNT ends are capped.

Synthesis of Bifunctional Linker

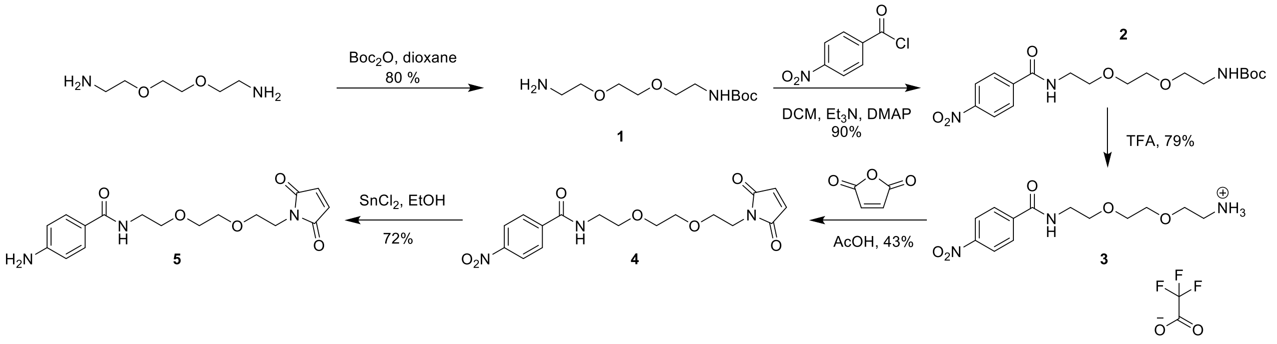

***tert*-Butyl (2-(2-(2-aminoethoxy)ethoxy)ethyl)carbamate 1.** Boc anhydride (2.45 g, 0.011 mol) was dissolved in 30 mL dioxane, and then added dropwise to 1,2-di(2-aminoethoxy)ethane (12.5 g, 0.085 mol) in 30 mL dioxane over two hours. The mixture was allowed to stir for a further 24 hours, after which the solvent was removed to yield a colourless liquid to which water (100 mL) was added. Three extractions with DCM (50 mL) were performed and the combined organic fractions were re-extracted with water (50 mL). The organic portion was dried over $MgSO_4$, and after filtration and solvent removal gave the product as a pale oil (2.18g, 80%). **^{1}H NMR** (400 MHz, $CDCl_3$) (ppm) 5.00 (1H, s, CON*H*), 3.54 (4H, s, $OC\textit{H}_2C\textit{H}_2O$), 3.50 – 3.44 (4H, m, $OC\textit{H}_2$), 3.27 (4H, m, $C\textit{H}_2N$), 2.02 (2H, br, $N\textit{H}_2$), 1.38 (9H, s, $C\textit{H}_3$); **^{13}C NMR** (100 MHz, $CDCl_3$) (ppm) 156.0 (*C*=O), 79.3 (*C*Me_3), 70.3 ($\textit{C}H_2$), 70.2 (3 x $\textit{C}H_2$), 40.4 (*C*NHCO, *C*NH_2), 28.4 (*C*H_3); **ESMS** m/z calc. for $[M+H]^+$ 249.2, found 249.2. See **Supplementary Fig. 11** for NMR spectra.

Boc-(2-(2-(2-(4-nitrobenzamido)ethoxy)ethoxy)ethylamine (2). 4-Nitrobenzoic acid (2.02 g, 12.1 mmol) was refluxed in thionyl chloride (25 mL) with catalytic DMF and DMAP for three hours under N_2. The solvent was removed *in vacuo* and DCM (25 mL) was used to dissolve the residue. Mono-Boc bis(amine) **1** (1.50 g, 6.04 mmol) was added, followed by triethylamine (1.5 mL), and the mixture was stirred overnight under nitrogen. The system was washed with 1M NaOH, 1M HCl, and brine (25 mL each), before being dried over $MgSO_4$, filtered, and subjected to solvent removal, to give a yellow residue containing some impurities (2.38 g, 100% if pure). It was found that purification could be achieved much more efficiently after the next step. **^{1}H NMR** (400 MHz, $CDCl_3$) (ppm) 8.20 (2H, d, ^3J = 2.7 Hz, Ar*H*), 7.91 (2H, d, ^3J = 2.7 Hz, Ar*H*), 6.97 (1H, br, CON*H*), 6.86 (1H, br, CON*H*), 3.64 – 3.54 (12H, m, $C\textit{H}_2$), 1.38 (9H, s, $C\textit{H}_3$); **^{13}C NMR** (100 MHz, $CDCl_3$) (ppm) 165.6 (Ar*C*ON), 156.0 (N*C*=OO), 149.4 (*C*NO_2), 140.2 (Ar*C*), 128.5 (Ar*C*), 123.6 (Ar*C*), 79.4 (*C*Me_3), 70.2 (4 x $\textit{C}H_2$), 40.3 ($\textit{C}H_2NH$), 40.0 ($\textit{C}H_2NH$), 28.4 (CH_3); **HR ESMS** m/z calc. for $[M+Na]^+$ 420.17412, found 420.17401. See **Supplementary Fig. 12** for NMR spectra.

N-(2-(2-(2-aminoethoxy)ethoxy)ethyl)-4-nitrobenzamide trifluoroacetate (3). Boc-amine **2** (2.38 g, 6.01 mmol) was stirred in dichloromethane (50 mL) with trifluoroacetic acid (25 mL) for three hours at room temperature. The volatiles were removed under vacuum and the residue was triturated with diethyl ether which was discarding. Remaining impurities were removed by azeotroping with dichloromethane to give the trifluoroacetate salt **3** as an orange oil (2.22 g, 79%). **^{1}H NMR** (400 MHz, CD_3OD) (ppm) 8.23 (2H, d, ^3J = 8.9 Hz, Ar*H*), 7.93 (2H, d, ^3J = 8.9 Hz, Ar*H*), 7.77 (1H, br, CON*H*), 3.62 (8H, m, $C\textit{H}_2$), 3.45 (2H, m, $C\textit{H}_2NHCO$), 2.77 (2H, m, $C\textit{H}_2NH_2$), 1.67 (3H, br, $N\textit{H}_3$); **^{13}C NMR** (100 MHz, CD_3OD) (ppm) 165.5 (Ar*C*ONH), 149.3 (*C*NO_2), 140.3 (*C*CONH), 128.4 (Ar*C*), 123.5 (Ar*C*), 73.1 ($C\textit{H}_2$), 70.3 ($C\textit{H}_2$), 70.0 ($C\textit{H}_2$), 69.5 ($C\textit{H}_2$), 41.5 ($CONHC\textit{H}_2$), 40.0 ($C\textit{H}_2NH_2$); **ESMS** m/z calc. for $[M+H]^+$ 298.1, found 298.1. See **Supplementary Fig. 13** for NMR spectra.

Maleimide 4. Amine **3** (0.10 g, 0.36 mmol) was added to acetic acid (10 mL), followed by maleic anhydride (0.040 g, 4.0 mmol). The mixture was stirred at room temperature for three hours, before being refluxed at 170 °C overnight. The solvent was removed and the product purified by silica gel column chromatography eluting with a gradient from 3 % to 10 % methanol in dichloromethane. The product emerged as the first band, and gave a yellow oil after solvent evaporation (0.055 g, 43 %). **^{1}H NMR** (400 MHz, $CDCl_3$) (ppm) 8.22

(2H, d, ^3J = 8.6 Hz, Ar*H*), 7.97 (2H, d, ^3J = 8.6 Hz, Ar*H*), 7.28 (1H, br, N*H*), 6.65 (2H, s, Mal*H*), 3.66 – 3.41 (12H, m, C*H*$_2$); **^{13}C NMR** (100 MHz, $CDCl_3$) (ppm) 170.7 (Mal*C*O), 165.5 (Ar*C*O), 149.5 (*C*NO_2), 134.2 (Mal*C*H), 128.4 (Ar*C*), 123.6 (Ar*C*), 70.1 (C*H*$_2$), 69.9 (C*H*$_2$), 69.5 (C*H*$_2$), 67.9 (C*H*$_2$), 40.0 (C*H*$_2$Mal), 37.1 (CONHC*H*$_2$); **HR-ESMS** m/z calc. for $[M+H]^+$ 378.12958, found 378.12958. See **Supplementary Fig. 14** for NMR spectra.

Aniline-maleimide 5. Maleimide **4** (20 mg, 5.3 x 10^{-5} mol) and tin dichloride dehydrate (54 mg, 2.12 x 10^{-4} mol) were dissolved in ethanol (2 mL). The mixture was heated to 70 °C for three hours. After cooling, saturated sodium hydrogen carbonate was added (1 mL), and the mixture was extracted with chloroform (3 x 5 mL). The combined organics were backwashed with saturated sodium hydrogen carbonate and brine (5 mL each), before being dried over magnesium sulphate, filtration, and solvent evaporation. The product was obtained as a yellow oil (13.3 mg, 72 %). **^{1}H NMR** (400 MHz, CD_3OD) (ppm) 7.54 (2H, d, ^3J = 7.6 Hz, Ar*H*), 6.64 (3H, m, Mal*H* + N*H*), 6.57 (2H, d, ^3J = 7.6 Hz, Ar*H*), 3.65-3.42 (6H, m, C*H*$_2$); **^{13}C NMR** (100 MHz, CD_3OD), 170.7 (Mal*C*O), 167.3 (Ar*C*ONH), 149.9 (*C*NH_2), 134.2 (Mal*C*H), 128.8 (Ar*C*), 123.8 (Ar*C*), 114.0 (Ar*C*), 70.1 (*C*H_2), 70.0 (*C*H_2), 69.8 (*C*H_2), 67.8 (*C*H_2), 39.5 (Mal*C*H_2), 37.1 (ArCONH*C*H_2); **HR-ESMS** m/z calc. for $[M+Na]^+$ 370.13734, found 370.13743. See **Supplementary Fig. 15** for NMR spectra.

Peptide Synthesis. Peptides were synthesised on a 0.5 mmol scale using rink amide or Wang resins as appropriate for an amide or acid group at the C-terminus respectively. The Fmoc-based synthesis was performed on the CEM Liberty microwave peptide synthesiser using acid-labile side chain protecting groups (Boc, Trt, Pbf). Quantities of Fmoc-X(PG)-OH peptide reagents were calculated through the provided software using the default coupling settings for each residue, and were dissolved in the indicated quantities of synthesiser-grade DMF, using sonication if necessary. Reagents were calculated similarly, using HBTU (0.5 M in DMF) as activator, DIPEA (2.0 M in NMP) as activator base, and piperidine (20%) and HOBt (0.1 M) in DMF for deprotection. The final Fmoc group was left on. After completion of synthesis, the resin was collected and washed thoroughly with DMF (3 x 15 mL), dichloromethane (3 x 15 mL), and diethyl ether (3 x 15 mL). TFA (10 mL) containing 2.5 % water and 2.5 % triisopropylsilane was added to cleave the peptide from the resin and remove the protecting groups. After allowing to react for an hour with occasional agitation, the mixture was filtered, with the solids being washed with further TFA (ca. 5 mL). The combined solutions were concentrated under a flow of nitrogen to less than 5 mL. Diethyl ether (20 mL) was then added to precipitate the crude peptide. About 0.1 g of crude product should be expected per peptide. Purification was achieved by reverse-phase HPLC using a C18 column (Phenomenex Synergi 4u Hydro-RP 80Å (100 x 4.6 µm, 4 micron) for analytical and Phenomenex Synergi 4u Hydro-RP 80Å (100 x 21.2 µm, 4 micron) for semi-prep scale). Initial screening was performed on the analytical scale, injecting 20 µL of sample at 2.0 mg/mL and running a gradient of 0 to 95 % MeCN in 95:5 H_2O:MeCN. Both solvent systems were spiked with 0.1 % formic acid and sonicated thoroughly to remove dissolved gases before use. Major peaks were collected as they eluted and analysed by MS to find the product – in all cases the largest peak. The gradient was optimised to ensure maximum separation of the product peak, and preparative separation of the peptide was performed using 1 mL injections at the maximum concentration of peptide in the starting solvent system. Multiple injections were required, and the collected product fractions were first concentrated on a rotary evaporator to remove organics, before the aqueous remainder was freeze-dried to give the pure product as a white to cream-coloured powder. See **Supplementary Fig. 16-19** for HPLC traces and MS.

Attachment of Maleimide to SWCNTs. Aniline **5** (0.180 g, 0.518 mmol) was sonicated with SWCNTs (31 mg, 2.59 mmol with respect to carbon atoms: 5 equivalents) in orthodichlorobenzene (25 mL) for 20 minutes. Isoamyl nitrite (84 µL, 73 mg, 0.622 mmol) was added and the mixture heated to 100 °C for 24 hours. Ethanol (75 mL) was added, and the mixture centrifuged. The supernatant was decanted, and 50 mL EtOH was added, followed by sonication. Centrifugation/supernatant removal was performed two more times with EtOH and two times with water. The SWCNTs were collected on a cyclopore membrane and dried under vacuum (51 mg). **Raman** A_D/A_G ratio of starting material = 4.85, product = 7.53. **TGA** (air) mass loss peaks 116, 361 °C (organics), 635 °C (CNTs), residue remaining = 0.2 wt%.

Attachment of Maleimide to PbO@SWCNTs. Aniline-maleimide **5** (29.5 mg, 8.5 x 10^{-5} mmol) was sonicated with PbO@SWCNTs (5.4 mg) in orthodichlorobenzene (5 mL) for 20 minutes. Isoamyl nitrite (13.7 µL, 12.0 mg, 0.102 mmol) was added and the mixture heated to 100 °C for 24 hours. Ethanol (35 mL) was added, and the mixture centrifuged. The supernatant was decanted, and 40 mL EtOH was added, followed by sonication. Centrifugation/supernatant removal was performed two more times with EtOH and two times with water. The SWCNTs were collected on a cyclopore membrane and dried under vacuum (9.1 mg). **Raman** A_D/A_G ratio of starting material = 7.44, product = 7.26. **TGA** (air) mass loss peak 133, 364, 487 °C (organics), 622 °C (CNTs), residue remaining = 14.9 wt%.

Attachment of Maleimide to BaI_2@SWCNTs. Aniline-maleimide **5** (29.5 mg, 8.5 x 10^{-5} mmol) was sonicated with BaI_2@SWCNTs (5.4 mg) in orthodichlorobenzene (5 mL) for 20 minutes. Isoamyl nitrite (13.7 µL, 12.0 mg, 0.102 mmol) was added and the mixture heated to 100 °C for 24 hours. Ethanol (35 mL) was added, and the mixture centrifuged. The supernatant was decanted, and 40 mL EtOH was added, followed by sonication. Centrifugation/supernatant removal was performed two more times with EtOH and two times with water. The SWCNTs were collected on a cyclopore membrane and dried under vacuum (10.9 mg). **Raman** A_D/A_G ratio of starting material = 4.96, product = 7.03. **TGA** (air) mass loss peak 117, 361, 482 °C (organics), 623 °C (CNTs), residue remaining = 17.0 wt%.

Attachment of Maleimide to Kr@SWCNTs. Aniline-maleimide **5** (29.5 mg, 8.5 x 10^{-5} mmol) was sonicated with Kr@SWCNTs (5.4 mg) in orthodichlorobenzene (5 mL) for 20 minutes. Isoamyl nitrite (13.7 µL, 12.0 mg, 0.102 mmol) was added and the mixture heated to 100 °C for 24 hours. Ethanol (35 mL) was added, and the mixture centrifuged. Incomplete separation was ameliorated by adding 10 mL of pentane (i.e. a less dense solvent). The supernatant was decanted, and 40 mL EtOH was added, followed by sonication. Centrifugation/supernatant removal was performed two more times with EtOH and two times with water. The SWCNTs were collected on a cyclopore membrane and dried under vacuum (10.0 mg). **Raman** A_D/A_G ratio of starting material = 6.88, product = 8.51. **TGA** (air) mass loss peak 112, 363, 480 °C (organics), 620 °C (CNTs), residue remaining = 13.7 wt%.

Further XRF details. The maps were of varying sizes, and hence took different amounts of time. The average time per map was slightly over one hour at 1 second per 1 μm^2 pixel. By reference to other measurements on the beamline, the photon flux was ca. 5 × 10^{11} ph sec^{-1} for 5 – 10 keV X-rays, and approximately half of that for 14 keV. Flux density was therefore on the order of 10^{11} ph μm^{-2} s^{-1} for 5 and 10 keV and half of that for 14keV. The detector was a 6-element silicon drift detector (from SGX) with a total area of 530 mm^2 and at distance of 70 mm was subtending ~0.1 sr. Synchrotron x-ray microprobes have detection limits in the sub-ppm regime, with the precise values depending on many factors including how the beamline has been setup and varies for the energy range covered by the elements observed. For a beam area of 5 μm^2 scanning a depth of 2 µm, 1 ppm corresponds to 10^{-17} g – i.e. the attogram regime. A conservative limit of detection would be 100 ag, which corresponds to ca. 10^5 heavy metal atoms. Given filled SWCNT diameters of 1 nm (as seen by HAADF-STEM), SWCNT lengths of 450 nm, and the Pb density of crystalline PbO, the limit of detection is in the region of 20 fully filled SWCNTs, or 150 SWCNTs filled at the levels seen here.

The spectra presented in Supplementary Figures 32 – 37 are taken from the maps, using single points of high intensity regions of the encapsulated material, and are indicative of the signal-to-noise which was obtained. In general, the XRF signals of the encapsulated agents were of similar magnitude to those the naturally present elements, and were 10-100 times more intense than the baseline. Much stronger signals can of course be seen taking the whole map average spectrum.

Supplementary References

1. Payne, D.J. et al. Experimental and theoretical study of the electronic structures of [small alpha]-PbO and [small beta]-PbO2. *J. Mater. Chem.* **17**, 267-277 (2007).
2. Kocbach, A. et al. Physicochemical characterisation of combustion particles from vehicle exhaust and residential wood smoke. *Part. Fibre Toxicol.* **3**, 1 (2006).
3. Zhou, W., Pennycook, S.J. & Idrobo, J.-C. Probing the electronic structure and optical response of a graphene quantum disk supported on monolayer graphene. *J. Phys. Condens. Matter* **24**, 314213 (2012).
4. The National Institute for Occupational Safety and Health (NIOSH), Lead compounds (as Pb). *http://www.cdc.gov/niosh/idlh/7439921.html* (accessed 10/05/2016).
5. The National Institute for Occupational Safety and Health (NIOSH), Barium (soluble compounds, as Ba). *http://www.cdc.gov/niosh/idlh/7440393.html* (accessed 10/05/2016).
6. Hulman, M., Kuzmany, H., Costa, P.M.F.J., Friedrichs, S. & Green, M.L.H. Light-induced instability of PbO-filled single-wall carbon nanotubes. *Appl. Phys. Lett.* **85**, 2068-2070 (2004).
